# Click Capture SELEX for the Identification of Click‐Modified Aptamers Targeting Small Molecules in Solution

**DOI:** 10.1002/chem.70932

**Published:** 2026-04-04

**Authors:** Philipp Menke, Carmelo Di Primo, Felix Bernhardt, Alea Schröder, Christian Renzl, Günter Mayer

**Affiliations:** ^1^ Life and Medical Science Institute (LIMES) University of Bonn Bonn Germany; ^2^ CNRS, INSERM, ARNA, UMR 5320, U1212 European Institute of Chemistry and Biology University of Bordeaux Pessac France; ^3^ Hochschule Bonn‐Rhein‐Sieg University of Applied Sciences Rheinbach Germany; ^4^ Center of Aptamer Research (CARD) University of Bonn Bonn Germany

**Keywords:** aptamer, CuAAC, method development, nucleobase modifications, SELEX

## Abstract

Capture SELEX is an in vitro selection method used to identify aptamers that bind small molecules in solution. The interaction of aptamers with their targets is primarily determined by supramolecular interactions, which are constrained by the chemical diversity of the canonical nucleobases. To overcome this limitation, the interaction properties of nucleic acid libraries can be expanded using nucleobase modifications. We developed click capture SELEX for the purpose of enriching chemically modified aptamers, also referred to as clickmers, which bind to small molecules. In this study, an indole‐modified DNA library was utilized to identify clickmers that bind specifically to kanamycin A. The click capture SELEX approach was employed to identify clickmers that bind small molecules in solution, thereby overcoming common limitations associated with using immobilized small target molecules in SELEX. The method described herein is generally adaptable to various small molecules.

## Graphical abstract

Using kanamycin A as a model target, we developed click‐capture SELEX to identify nucleobase‐modified aptamers capable of binding small molecules in solution. This approach enables flexible incorporation of nucleobase modifications via CuAAC, allowing the chemical properties of the aptamer to be tailored to the target molecule.

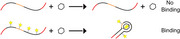

## 1 Introduction

Aptamers are short single‐stranded oligonucleotides that fold into distinct 3D structures and specifically bind to various target molecules, such as cells, proteins, and small molecules [1]. These molecules are identified through an in vitro selection method termed Systematic Evolution of Ligands by Exponential Enrichment (SELEX) from extensive nucleic acid libraries [2]. Aptamers bind their target molecules by means of supramolecular interactions, including hydrogen bonding and electrostatic forces [3]. However, the limited chemical diversity of the canonical nucleobases imposes constraints on the range of targets for which aptamers can be identified [4]. To overcome this limitation, modified nucleobases, artificial base pairs that expand the genetic alphabet or xeno‐nucleic acids (XNA) with modified backbones can be incorporated into nucleic acid libraries [4, 5, 6, 7, 8]. The advent of nucleobase modifications facilitated access to hitherto unreachable target molecules and augmented the success rates of SELEX [4]. This phenomenon can be attributed to enhanced target‐aptamer interactions and broader structural diversity [9, 10]. The click SELEX method [11] involves the replacement of thymidine with 5‐ethynyl‐2’‐deoxyuridine (E‐dU). E‐dU contains a terminal alkyne group that can be modified with azides via copper‐catalyzed azide‐alkyne cycloadditions (CuAAC) [11, 12]. The E‐dU is well tolerated by most common polymerases and is commercially available [13]. In principle, the introduction of any chemical group into a DNA strand is possible, provided the corresponding azide is stable under the reaction conditions and, once incorporated, also tolerated as a template during PCR. Aptamers identified by click SELEX are designated as clickmers. Clickmers have been identified for various proteins using different modifications [11, 14, 15]. Moreover, the application of clickmers has been extended to 𝚫^9^‐tetrahydrocannabinol (THC) [16]. However, this click SELEX experiment employed (–)‐𝚫^9^‐tetrahydrocannabinolic acid (THCA) that was chemically modified for immobilization on magnetic particles. The resultant clickmers exhibited binding affinity to both the THCA and the THC, although with a reduced affinity to the latter [16]. This finding can be attributed to the immobilization strategy, which created neo‐epitopes and modified the electron distribution within the target molecule when compared to THC in solution. The application of (click) SELEX to immobilized small molecules also poses a substantial risk of selecting aptamers that bind to the small‐molecule‐bead complex rather than the small molecule in solution [17].

In order to address these challenges, the click SELEX procedure was adapted to capture SELEX processes [18]. In the capture SELEX process, the nucleic acid library is immobilized on a capture oligo (CapO) that is attached to a solid support through embedded docking sequences. The target molecules are introduced into the solution, thereby interfering with the equilibrium between sequences that are immobilized on the solid support and those that are free in solution. Sequences that bind to a target molecule undergo structural changes that prevent their rehybridization to the CapO, allowing them to be recovered and amplified by PCR. In this study, we delineate the successful development of click capture SELEX, a methodology that facilitates the identification of clickmers targeting small molecules in solution. We selected kanamycin A as a model target because it is a frequently utilized molecule in capture SELEX, yielding RNA or DNA aptamers [19, 20, 21]. We identified the clickmer CP5, which demonstrated a high degree of binding affinity and specificity for kanamycin A in a click‐in dependent manner.

## 2 Results and Discussion

**FIGURE 1:**
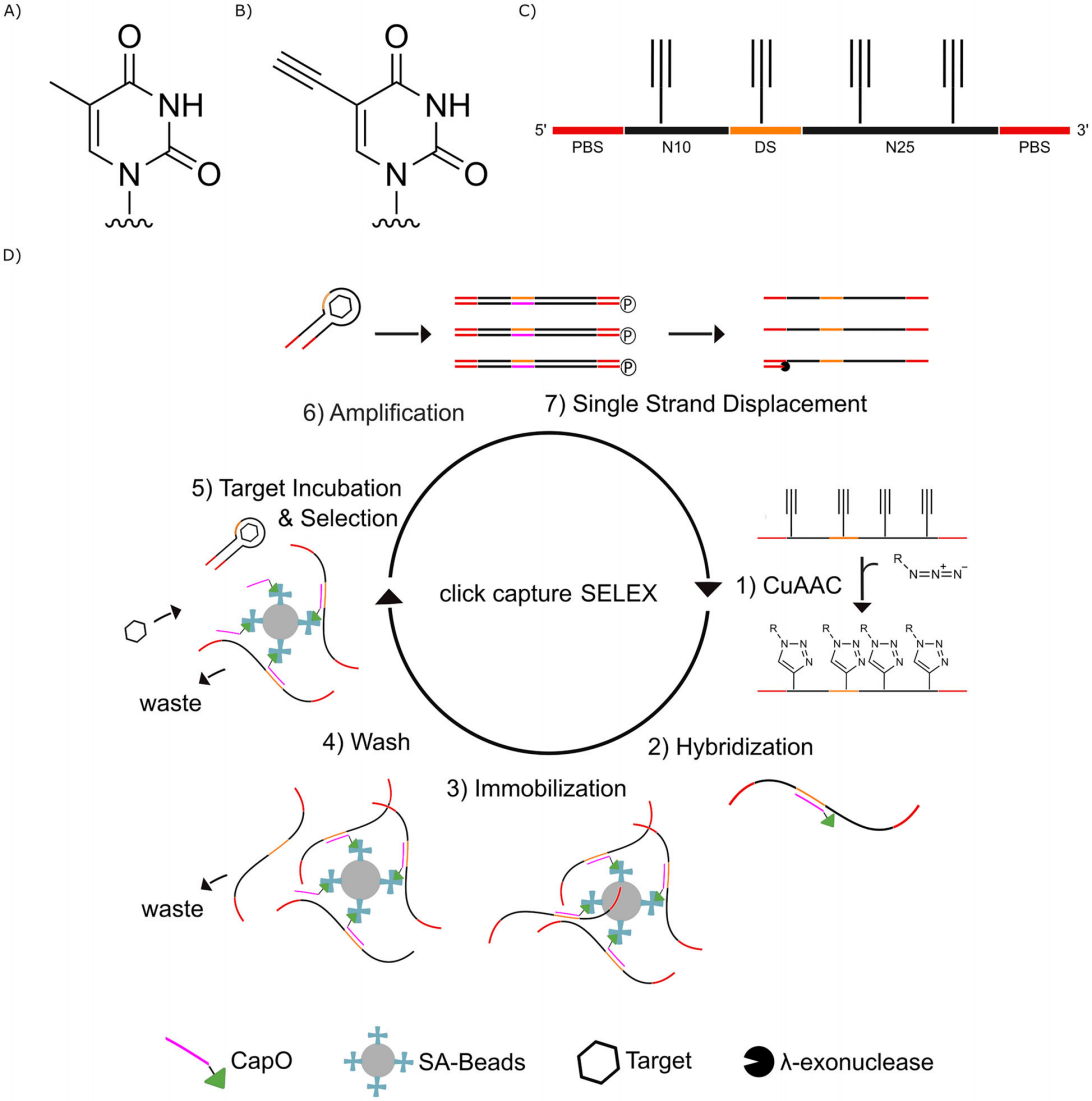
Schematic concept of the click capture SELEX (A) Chemical structures of thymidine (dT) and (B) 5‐Ethynyl‐2’‐deoxyuridines (E‐dU). (C) The SELEX library CP‐L used in this study comprises two primer‐binding sites and a constant docking sequence (DS), flanked by 10‐ and 25‐nucleotide‐long random regions (N10 and N25). In the random region all dTs are replaced with E‐dU (D) Schematic representation of the click capture SELEX. 1) Functionalization of the library via copper‐catalyzed azide‐alkyne cycloaddition (CuAAC). 2) Hybridization of the library to a biotinylated capture oligo (CapO) complementary to the DS of the library. 3) Immobilization of the library capture oligo complex on streptavidin‐coated magnetic beads (SA‐beads). 4) Removing weakly‐ and unbound sequences via several washing steps. 5) Addition of the target to the immobilized sequences, followed by the separation and selection of the recovered sequences. The target binding initiates conformational changes in the DNA, preventing re‐hybridization to the SA‐beads. 6) Amplification of the recovered sequences via PCR. 7) λ‐exonuclease digestion of the phosphorylated reverse strand. The remaining forward strand is used in the next selection cycle.

To establish click capture SELEX, a DNA library, designated CP‐L, was designed in which each thymidine (dT) was replaced by E‐dU (Figure 1A, B). CP‐L contains two primer‐binding sites at the 5’ and 3’ ends, as well as a docking sequence (DS) flanked by two random regions with 10 and 25 random nucleotides, respectively (Figure 1C). Placing the DS within the random region facilitates ligand‐induced refolding of the clickmer in its binding domain [18]. Alternatively, the DS can also be integrated into one of the primer‐binding sites of the SELEX libraries. However, this design limits the structural space to sequences that form a stem loop with their two primer‐binding sites after target binding. Placing the DS within the random region allows for more flexible target‐induced refolding. The design of the primer‐binding sites of CP‐L is related to previous library designs used for successful click‐SELEX experiments [11]. The 12‐nt DS design is adapted from Legen et al. and Stoltenburg et al. [20, 22]. In accordance with the findings of previous studies, a statistical number of 4 E‐dUs per sequence was selected. This number was determined to be optimal for amplification efficiency and enrichment of binding species [23]. In the initial step of the procedure (Figure 1D), CP‐L is modified via CuAAC with an azide of choice. Subsequently, the biotinylated capture oligo (biotin‐CapO) is hybridized to the library, and the CP‐L:biotin‐CapO complexes are immobilized on streptavidin‐coated magnetic beads (SA‐beads). Sequences that are not or weakly bound to the SA‐beads are removed through washing steps. After incubating these beads with the target molecule, the sequences that bind to the target are separated from the beads and amplified by PCR. Subsequent to PCR, the reverse strand is subjected to digestion by λ‐exonuclease, and the remaining forward strand is used in the next selection cycle. All oligodeoxynucleotide sequences used in this study are listed in Table S1 in the Supporting Information.

**SCHEME 1:**
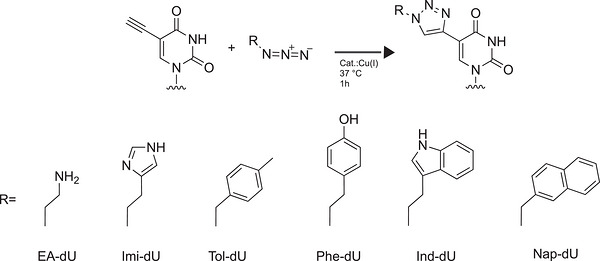
CuAAC Reaction Scheme with the chemical structures of the nucleobase modifications used in this study (R). EA‐dU = 2‐azidoethanamine; Imi‐dU = 4‐(2′‐azidoethyl)‐1*H*‐imidazole; Tol‐dU = 1‐azidomethyl)‐toluol; Phe‐dU = 4‐(2’‐azidoethyl)phenol; Ind‐dU = 3‐(2′‐azidoethyl)‐1*H*‐indole; Nap‐dU = 1‐(azidomethyl)naphthalene.

The configuration of the docking sequence is crucial for the differentiation of binding candidate sequences. The process of target‐induced refolding of the putative binding sequences interferes with bead hybridization, thereby maintaining bound sequences in solution. In order to provide support for this hypothesis, we decided to maintain one E‐dU in the DS to promote ligand‐induced refolding at this position. However, it is important to note that alterations in the DNA sequence can potentially influence the hybridization kinetics of the DS with the CapO. Consequently, we employed biolayer interferometry (BLI) to assess the impact of nucleobase modifications in the docking sequence on its interaction with the CapO. We modified the docking sequence (CP‐DS) with six different entities (2‐azidoethanamine (EA‐dU), 4‐(2′‐azidoethyl)‐1*H*‐imidazole (Imi‐dU), 1‐azidomethyl)‐toluol 1‐azidomethyl)‐toluol (Tol‐dU), 4‐(2’‐azidoethyl)phenol (Phe‐dU), 3‐(2′‐azidoethyl)‐1*H*‐indole (Ind‐dU) and 1‐(azidomethyl)naphthalene (Nap‐dU)) (Scheme 1) and measured their binding kinetics to the CapO (Figure S1, Supporting Information). The click efficiency for these and other oligodeoxynucleotides utilized in this study was analyzed via LC‐MS (Figures S2–S34, Supporting Information).

**FIGURE 2:**
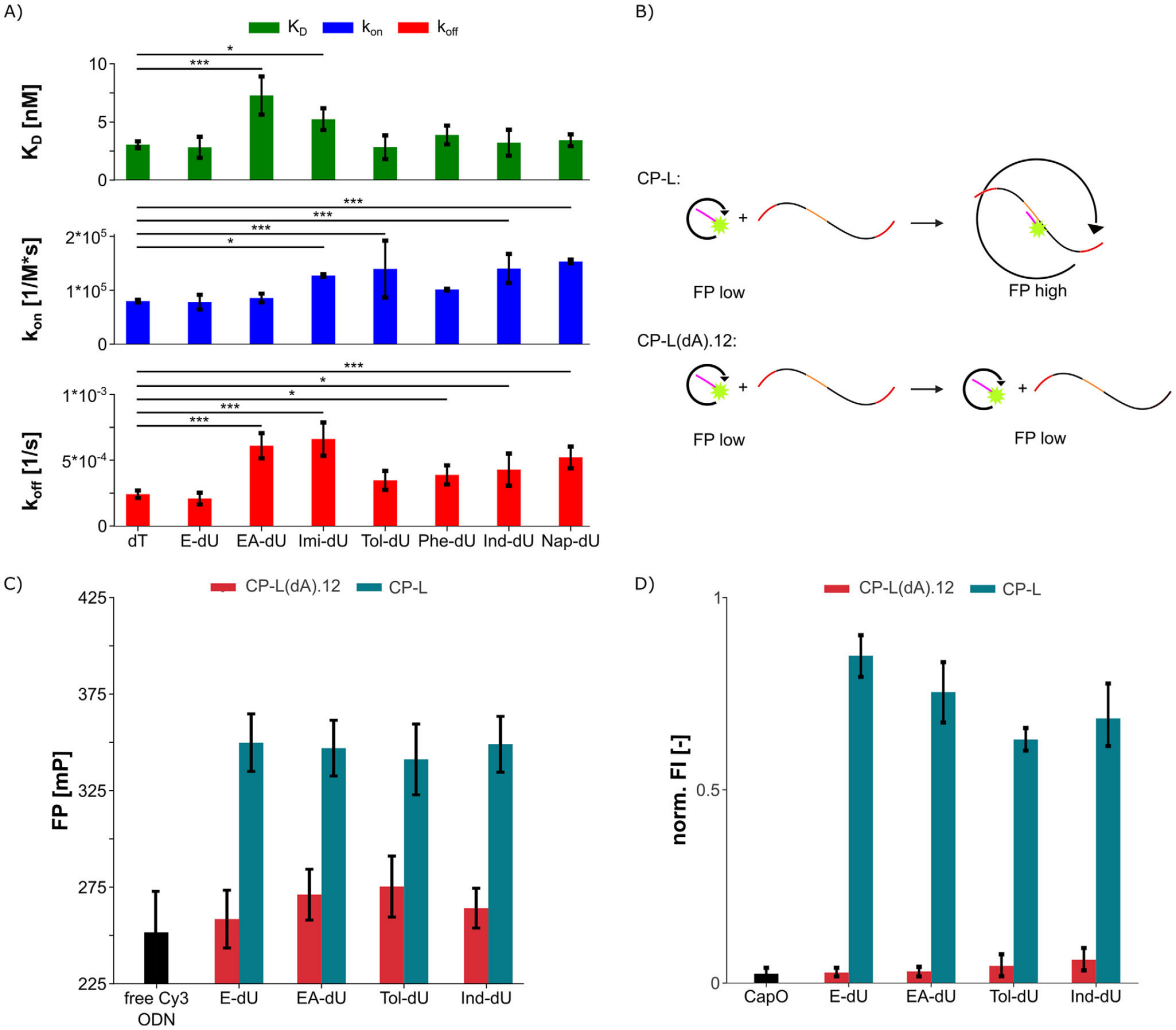
Evaluation of the influence of nucleobase modifications on the hybridization and immobilization during the click capture SELEX. (A) Study of the binding kinetics of the docking sequence of CP‐L (CP‐DS) and its complementary strand (CP‐DS‐Rev) using biolayer interferometry. dT, E‐dU, EA‐dU, Imi‐dU, Tol‐dU, Phe‐dU, Ind‐dU, and Nap‐dU indicate modifications. (N = 3 in duplicates, mean ± sd, ONE‐Way ANOVA followed by Tukey's post‐hoc test). (B) Schematic representation of the fluorescence polarization assay. FP: fluorescence polarization (C) Hybridization efficiency analysis of a with EA‐dU, Tol‐dU, and Ind‐dU functionalized CP‐L SELEX library in comparison to CP‐L(dA).12 bearing the same modifications via FP. (N = 2 in triplicates, mean ± ci95%). (D) Immobilization efficiency of CP‐L with various chemical modifications on streptavidin‐coated magnetic beads (SA‐beads) relative to CP‐L(dA).12. norm. FI: normalized fluorescence intensity (N = 2 in triplicates, mean ± ci95%).

The BLI analysis (Figure 2A) shows that substituting dT with E‐dU does not alter the binding kinetics of the DNA duplex. The incorporation of EA‐dU and Imi‐dU modifications results in a modest increase in the dissociation constant (K_D_). Both modifications increase the dissociation constants (k_off_) relative to the native duplex. The Imi‐dU modification has been shown to increase the association constant (k_on_). The incorporation of Ind‐dU and Nap‐dU modifications results in an increase in k_off_. However, this destabilizing effect is counterbalanced by an increase in k_on_, resulting in a K_D_ analogous to that of unmodified DNA. The value of the k_off_ of Phe‐dU modified DS also increases. However, the destabilizing effect does not impact the K_D_. The k_on_ of Tol‐dU modified DNA also increases. In a manner analogous to Phe‐dU, this effect does not impact the K_D_. Overall, the affinities for CapO across all modifications are in the low nanomolar range. All analyzed nucleobase modifications allow stable hybridization of the DS with CapO, thereby rendering them suitable for capture SELEX. Details on the kinetic parameters are provided in Table S2 of the Supporting Information.

Subsequently, the hybridization efficiency of nucleobase‐modified libraries (CP‐L) was evaluated using a fluorescence polarization (FP) assay (Figure 2B). To this end, we hybridized the library, which contained various modifications to a CapO labeled with a Cy3 fluorophore and shortened to nine nucleotides (Cy3‐ODN). In solution, the non‐hybridizing Cy3‐ODN rotates rapidly. When the oligo is exposed to polarized light, a significant portion of the FP is lost, leading to the emission of unpolarized light by the fluorophore. The binding of Cy3‐ODN to CP‐L results in a decrease in the rotational rate of the complex. When exposed to polarized light, the fraction of emitted polarized light increases. As demonstrated in Legen et al. [22]., the presence of shorter oligos is associated with disparities in the FP.

We used EA‐dU, Tol‐dU, and Ind‐dU as representative candidates to modify CP‐L, each exhibiting distinct effects on duplex stability (Figure 2A). All modified CP‐Ls showed an increase in FP values, similar to those observed in the CP‐L without modifications (Figure 2C). Conversely, no augmentation was detected with CP‐L(dA).12, wherein the DS was substituted by 12 adenosine nucleotides. The Cy3‐ODN cannot hybridize to the CP‐L(dA).12 and stays free in solution. Therefore, the FP remains low.

Next, we investigated the efficiency with which the modified libraries could be immobilized on magnetic beads. We hybridized the CP‐Ls with the different modifications (EA‐dU, To‐dU, and Ind‐dU) to biotin‐CapO and incubated the CP‐L:biotin‐CapO complex with SA‐beads. After incubation, the supernatant was removed and the remaining immobilized sequences were recovered by heat denaturation and quantified using Quant‐iT OliGreen (Figure 2D). It was observed that all modified CP‐L exhibited a lower immobilization efficiency in comparison with unmodified CP‐L (E‐dU). However, owing to the absence of normal data distribution, we were unable to test for statistical significance. Minimal nonspecific binding of CP‐L(dA).12 was observed. We compared the immobilization efficiency of the CP‐L library and that of the CP‐L(dA).12 library, which bears the same modifications. The modified CP‐L(dA).12 libraries exhibited no immobilization on the SA‐beads, thereby confirming that immobilization occurs exclusively through the DS strand hybridized to the CapO on the SA‐beads. Despite slightly diminished immobilization efficiency exhibited by modified CP‐L, all examined modifications showed high sequence loading on the beads.

**FIGURE 3:**
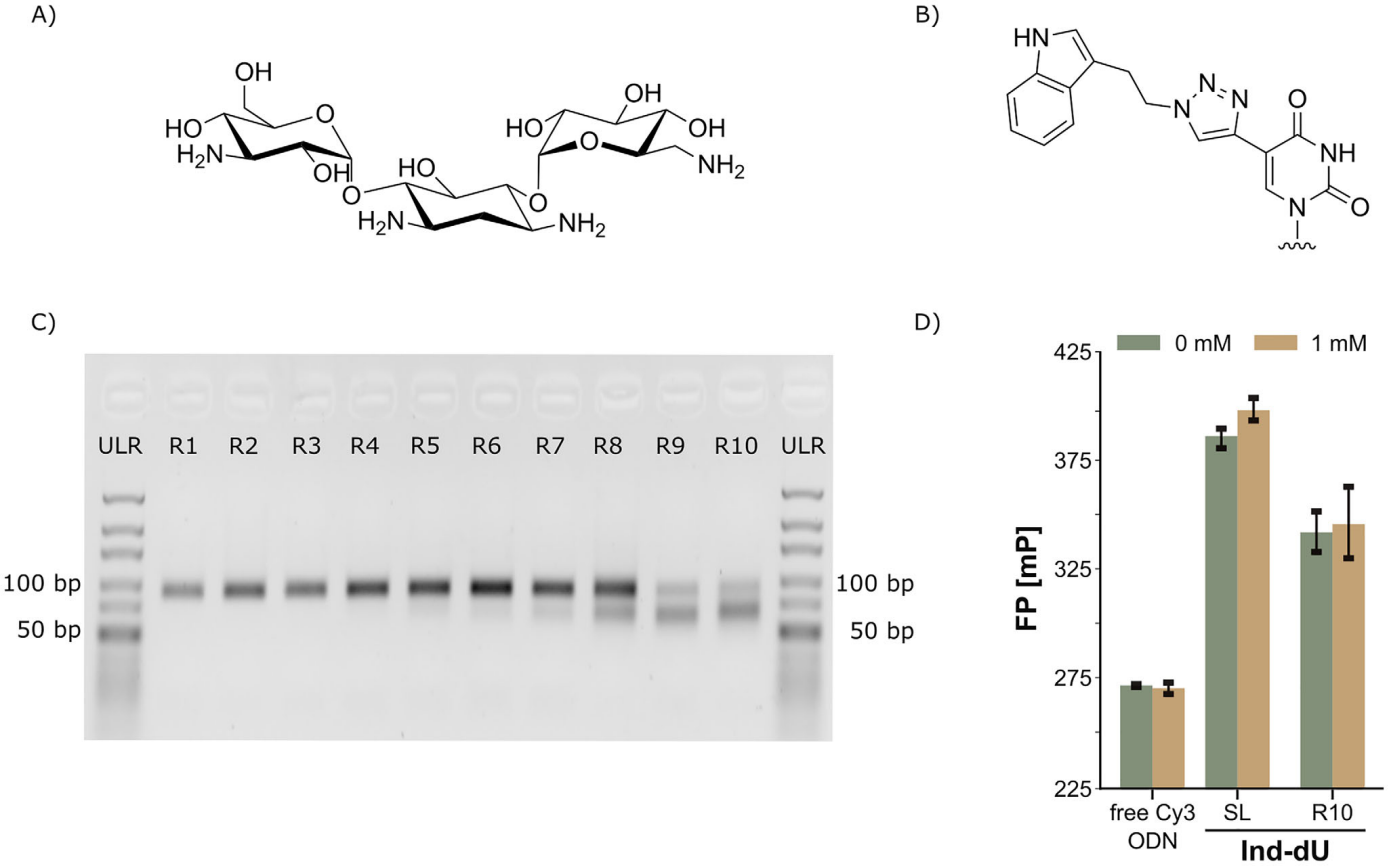
Click capture SELEX to identify clickmers that bind to kanamycin A. (A) Chemical structure of kanamycin A. (B) Chemical structure of 3‐(2‐azidoethyl)‐1H‐indole‐uridine (Ind‐dU). (C) PCR products of the ten selection rounds. ULR: Ultra Low Range Ladder from Thermo Fisher; R1 to R10: selection round 1 to 10; bp: basepair. (D) Binding of round ten of the selection to kanamycin A [0 and 1 mM] compared to the starting library, measured by fluorescence polarization. SL: starting library; E‐dU, Ind‐dU, indicate modifications; FP: Fluorescence polarization. (N = 2 in triplicates, mean ± ci95%).

Having established the individual steps of the click capture SELEX, the selection scheme was applied to kanamycin A (Figure 3A). Kanamycin A was chosen as a model target molecule because aptamers that bind to it are described in literature [19, 20, 21], rendering it a suitable target molecule. We used CP‐L modified with Ind‐dU (Figure 3B), since Ind‐dU‐modified libraries have been successfully employed in previous click SELEX experiments [11, 14, 15]. Furthermore, the increased kinetic rate constants observed with indole (Figure 2A) can lead to more dynamic association and dissociation from the SA‐beads, increasing the likelihood of finding and binding to a target molecule. Nap‐dU has been shown to have similar kinetic rate constants; however, in comparison to Ind‐dU, Nap‐dU lacks a hydrogen bond donor that could directly interact with the functional groups of kanamycin A. To this end, ten selection rounds were performed using decreasing concentrations of kanamycin A in the elution step to increase stringency (Table S3, Supporting Information). However, after six SELEX cycles, an unanticipated by‐product emerged following the PCR amplification and subsequently became the predominant product in selection cycle 10 (Figure 3C). The formation of by‐products is a common occurrence during SELEX experiments [24]. Its occurrence frequently impacts the enrichment of binding species [24]. Accordingly, the SELEX was terminated after 10 rounds, and the enrichment of kanamycin‐binding sequences was analyzed using FP assays (Figure S35, Supporting Information). A comparison was made of the FP‐values of the starting library (SL) and the library after ten selection rounds (R10) in the presence and absence of kanamycin A (Figure 3D). However, the FP values for both samples were not statistically significant at any target concentration. It is noteworthy that the FP‐values obtained from R10 are lower than those from the SL. This finding contradicts previous observations, which indicated that enriched libraries generally exhibited stronger binding affinity to CapO [22]. This finding suggests that sequences in R10 are enriched for those with a reduced affinity for CapO, either due to acquired mutations in the DS or due to the presence of distinct structures within the modified library that impede the interaction between the DS and CapO.

**FIGURE 4:**
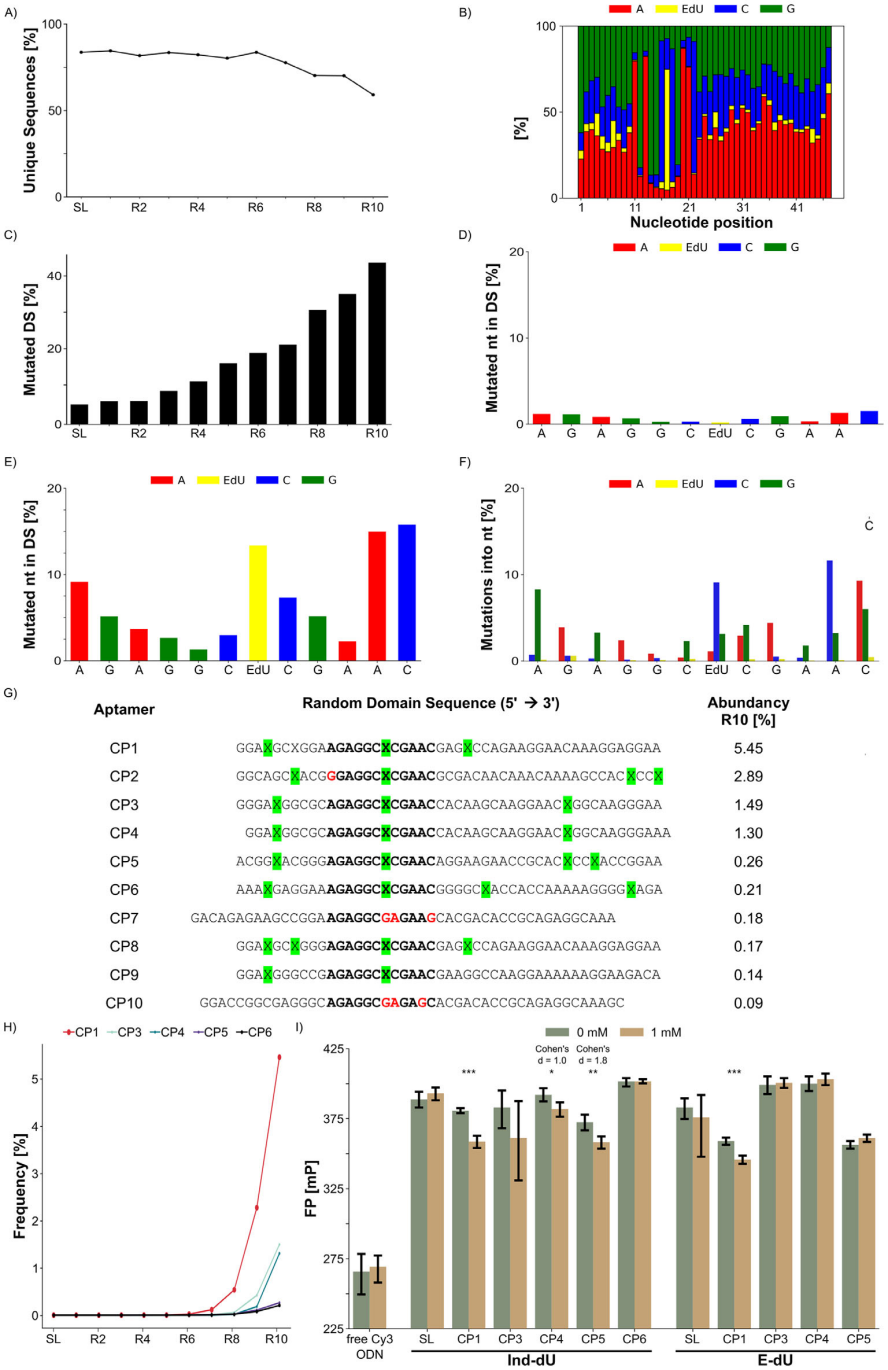
NGS analysis and identification of kanamycin A binding sequences. (A) Proportion of unique sequences over the selection rounds. (B) Nucleotide distribution from the random region of the round ten SELEX library. (C) Number of mutated docking sequences (DS) over the selection rounds. (D) Mutation analysis of each position of the docking sequence from the starting library. (E) Mutation analysis of each position of the docking sequence from the round ten SELEX library. Mutation frequency at which mutations changed the original nucleotide in the docking sequence from the starting library at the denoted position. (F) Mutation frequency with which mutations converted the original nucleotide from the docking sequence from the round ten SELEX library into the denoted nucleotide. (G) Top ten most abundant sequences after ten selection rounds. The docking sequence is highlighted in bold, mutations in the docking sequence are written in red and the possible sites for mutations (X) are highlighted in green. R10: selection round ten. (H) Evolution of the five most abundant sequences without a mutation in the docking sequence over the ten selection rounds. (I) Binding of the five most abundant sequences from the click capture SELEX (CP1, CP3, CP4, CP5 and CP6) to kanamycin A [0 and 1 mM] measured by fluorescence polarization (FP). SL: starting library; E‐dU, Ind‐dU, indicate modifications (At least N = 2 in triplicates, mean ± ci95%, unpaired t‐test).

Subsequently, next‐generation sequencing (NGS) was performed to analyze the DNA populations of the enriched libraries (Table S4, Supporting Information). Sequence analysis indicates that even after 10 selection rounds, the library is predominantly comprised of unique sequences (59%, Figure 4A) and there is a decrease in the number of E‐dU nucleotides (Figure 4B) in comparison to the SL (Figure S36A, Supporting Information). An accumulation of mutations was observed in the DS (Figure 4C), with one or more mutations (Figure S36B, Supporting Information). Additionally, a shift in the initial position of the DS was observed (Figure S36C). These mutations likely decrease the stability of the DS‐CapO complex and might be responsible for the lower FP values of the R10 library (Figure 3D). In contrast, the starting library contains only a low fraction of mutated nucleotides in the DS, with the number of mutations increasing in frequency toward the 3’ end of the DS (Figure 4D). This phenomenon is also reflected by the DNA population from selection round 10 (Figure 4E). Additionally, an elevated mutation rate was observed in two nucleotides downstream and the nucleotide preceding the E‐dU. The purine nucleobases in the DS were found to predominantly mutate through purine‐purine exchanges, with a single exception. The adenine at position eleven predominantly mutates into a cytosine rather than a guanine (Figure 4F). Additionally, the SL exhibits the same mutation at this specific position (Figure S3D, Supporting Information). However, the pyrimidine nucleobases exhibit distinct patterns of mutagenesis, resulting in different types of nucleotides. E‐dU predominantly mutates to cytosine, an observation that is consistent with the anticipated order of replacement of a pyrimidine base by another pyrimidine base. Conversely, cytosines are predominantly substituted by adenine or guanine bases. These data corroborate the findings of Pfeiffer et al. [25], which demonstrated that E‐dU imposes elevated error rates during PCR. The E‐dU in the DS is frequently mutated, a process that destabilizes the CapO:Library complex. This, in turn, explains the lower binding intensity of the DNA from selection round ten compared to the starting library (Figure 3D). In our previous capture, SELEX approaches using unmodified RNA libraries, the DS was more conserved, with less than 5% mutations in the DS over the selection rounds [22, 26]. During the SELEX process, pyrimidine bases are more prone to mutate into purine bases, while E‐dU mutates into cytosine and cytosine into either adenine or guanine. The observed decline in E‐dU within the random region, from 10% in the SL to 4% in the library after ten rounds of selection, can be attributed to the mutational rates of the docking sequence. A substantial proportion of the ten most abundant sequences contain at least one E‐dU in their random region, and the six most abundant sequences have at least two E‐dUs (Figure 4G). The ten most abundant sequences constitute 12.2% of the library population from selection round 10, while the most abundant sequence (CP1) comprises 5.5% of the library. It is notable that CP3, CP4 and CP5 exhibit E‐dU at two identical positions. Three of the top ten sequences contained mutations in their DS, and they were subsequently excluded from further analysis. The five most abundant sequences, devoid of mutations in the DS, undergo enrichment following selection round 6 (Figure 4H); nevertheless, their abundance remains minimal (< 6%, Figure 4G). We analyzed these sequences for their binding properties to kanamycin A using FP assays (Figure 4I). To identify a sequence that can bind to kanamycin A in click dependency, we measured the binding ability of them in their indole‐modified and unmodified (E‐dU) forms. Based on these results, the five sequences can be arranged into three categories. In the case of CP6, no binding to kanamycin A was observed. The second category comprises click‐independent binders, which include CP1. This sequence binds to kanamycin A irrespective of the clicked‐in modification. The third category encompasses the click‐dependent binders, which include CP4 and CP5. CP3 might also fall into this group; however, due to its high error margin, we were unable to determine its significance. CP4 and CP5 have been identified as candidates that bind kanamycin A in their Ind‐dU‐modified states. Subsequent studies focused on CP5, based on statistical analysis using Cohen's d effect size [27], in which CP5 (Cohen's d = 1.8) outcompetes CP4 (Cohen's d = 1.0). Compared with other capture SELEX trials [22, 28], the observed sequence diversity was still elevated. Concurrently, the abundance of kanamycin A binding sequences is found to be minimal. The low proportion of binding sequences likely explains the absence of a detectable signal in the binding assay of the libraries following the selection round ten (Figure 3D). Notwithstanding the low proportion of binding sequences in the selected libraries, these sequences could be identified through the use of deep sequencing and sequence analysis.

**FIGURE 5:**
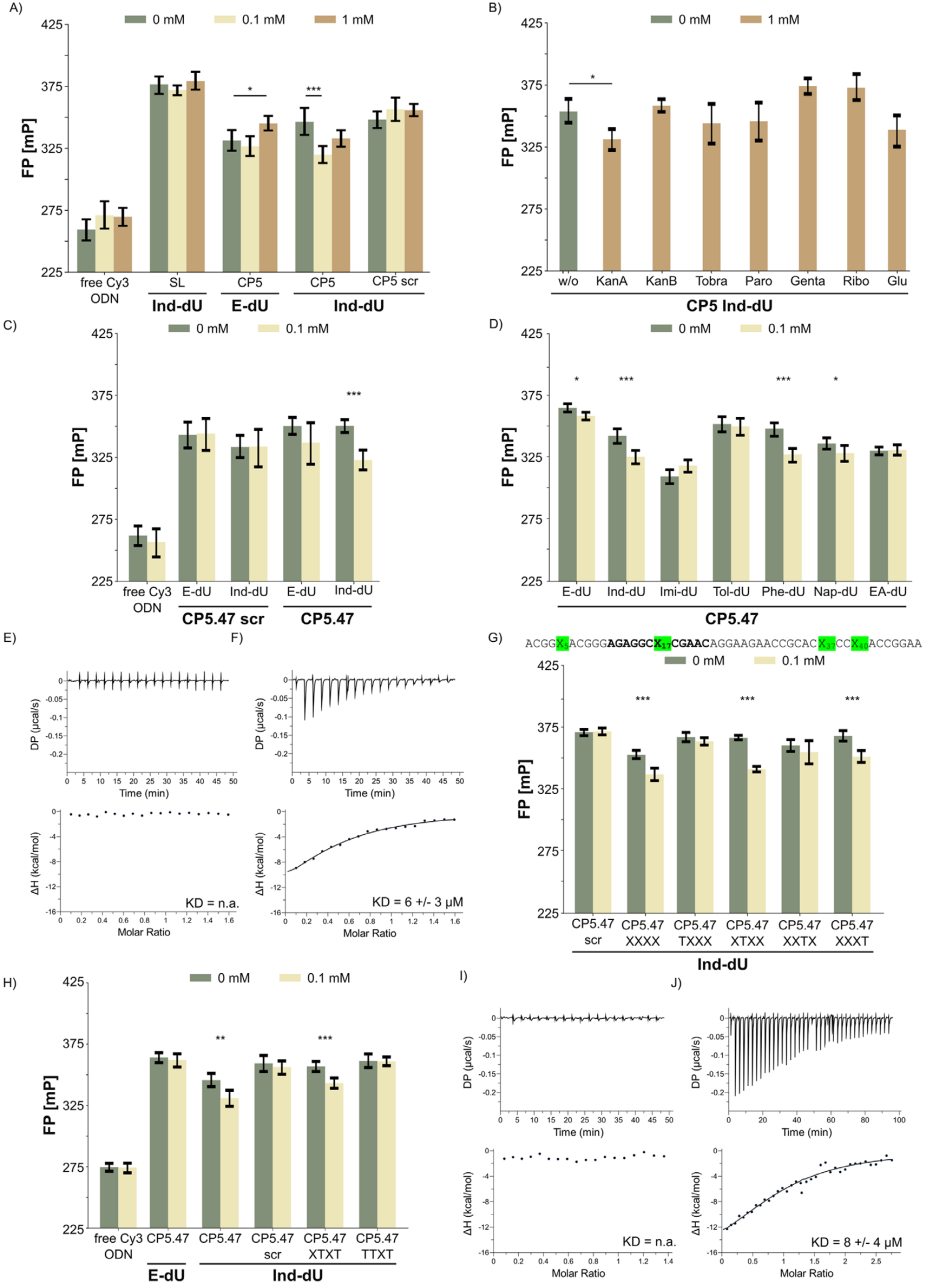
Characterization of the clickmer CP5. (A) Binding of CP5 to kanamycin A [0 mM, 0.1 and 1 mM] measured by FP. SL: starting library; E‐dU, Ind‐dU, indicate modifications; CP5scr: control sequence with the same nucleotides as CP5 but in a different distribution. (N = 5 in technical triplicates, mean ± ci95%, ONE‐Way ANOVA followed by Tukey's post‐hoc test) (B) Specificity analysis of CP5 measured by FP. KanA: Kanamycin A; KanB: Kanamycin B; Tobra: Tobramycin; Paro: Paromomycin; Genta: Gentamicin; Ribo: Ribostamycin; Glu: D‐(+)‐Glucose. (at least N = 4 in triplicates, mean ± ci95%, ONE‐Way ANOVA followed by Tukey's post‐hoc test) (C) Binding ability of CP5.47 to kanamycin A. (N = 5 in triplicates, mean ± ci95%, unpaired t‐test) (D) Replacement of Ind‐dU in CP5 with other modifications. (N = 5 in triplicates, mean ± ci95%, unpaired t‐test). (E‐F) Isothermal titration calorimetry (ITC) experiments of CP5.47 in its E‐dU (E) and Ind‐dU‐modified (F) form with kanamycin A. K_D_‐value of Ind‐dU CP5.47 and kanamycin A is 6 ± 3 µM. (G) Identification of In‐dU‐modified positions contributing to kanamycin A binding. E‐dU (X) was replaced by thymine (T). (N = 4 in technical triplicates, mean ± ci95%, unpaired t‐test) (H) FP assay of double‐modified CP5.47 (CP5.47 XTXT). (N = 5 in triplicates, mean ± ci95%, unpaired t‐test) (I‐J) ITC of CP5.47 TTXT (I) and CP5.47 XTXT (J) with kanamycin A. K_D_‐value of Ind‐dU CP5.47 XTXT and kanamycin A is 8 ± 4 µM.

To further characterize CP5, a nonbinding scrambled version of CP5 (CP5 scr) was generated to assess the sequence dependence of kanamycin A binding (Figure 5A). Next, the specificity of CP5 was analyzed (Figure 5B), and its interaction with structurally related targets, including kanamycin B, tobramycin, paromomycin, gentamicin, ribostamycin and D‐glucose was compared (Scheme S1, Supporting Information). The FP‐assays revealed specific binding of CP5 to kanamycin A (Figure 5B) and no significant interaction was detected using related molecules. Kanamycin B differs from kanamycin A only at the C‐2″ position of one sugar moiety. At this position, kanamycin A has a hydroxyl group and kanamycin B has an amino group, similarly to tobramycin and gentamicin. These data suggest that the hydroxyl group at this position directly interacts with the clickmer.

We truncated CP5 to 47 nucleotides (CP5.47), essentially by removing the primer‐binding sites, and observed binding of CP5.47 to kanamycin A (Figure 5C). Analogous to the full‐length clickmer, Ind‐dU‐dependent binding of CP5.47 was also maintained. The binding affinity of CP5.47 to Cy3 ODN was not affected by the modification (Figure S37, Supporting Information).

In order to analyze the importance of Ind‐dU for kanamycin A binding, it was replaced in CP5.47 with Tol‐dU, Imi‐dU, Phe‐dU, Nap‐dU and EA‐dU, respectively, and the ability of these modifications to mediate binding to kanamycin A was then tested (Figure 5D). The Phe‐dU modification was found to support CP5's binding to kanamycin A. Indole and phenol both contain an aromatic ring and a hydrogen‐bond donor positioned outside the aromatic system, which could contribute to the binding of kanamycin A. However, it remains unclear whether these side chains directly interact with kanamycin A or whether they stabilize the folding of the clickmer required for binding. In these experiments, both the E‐dU and the Nap‐dU variants demonstrated a decreased FP signal in the presence of kanamycin A. However, the signal difference obtained in the presence or absence of kanamycin A was found to be smaller compared to the ones obtained from the Ind‐dU‐modified CP5.47 (p = 0.04, unpaired t‐test). To ascertain in‐depth interaction characteristics, we performed isothermal titration calorimetry (ITC) (Figure 5E). In these experiments, no interaction of kanamycin A with E‐dU CP5.47 was detected. Conversely, the Ind‐dU‐modified CP5.47 demonstrated binding affinity for kanamycin A with a K_D_ of 6 ± 3 µM (Figure 5F). We employed an additional titration assay, based on FP (Figure S38, Supporting Information), which confirmed the data obtained by the ITC.

CP5 contains four Ind‐dU modifications. Next, the importance of each position for kanamycin A binding was systematically examined. Four CP5.47 derivatives were designed, with one modification being substituted with dT (Figure 5G). Interaction analysis revealed that those sequences in which E‐dU_5_ (CP5.47 TXXX) and E‐dU_37_ (CP5.47 XXTX) were replaced with dT lost their ability to bind to kanamycin A. In contrast, the replacement of E‐dU_17_ (CP5.47 XTXX) and E‐dU_40_ (CP5.47 XXXT) with dT still allows binding to kanamycin A. Importantly, a variant of CP5 in which only E‐dU_5_ and E‐dU_37_ are modified with indole also retained binding properties (CP5.47 XTXT) (Figure 5H), indicating that these two indole modifications are sufficient for kanamycin A interaction. In accordance with the variant CP5.47 TXXX (Figure 5H) the interaction with kanamycin A is lost in CP.47 XTXT when E‐dU_5_ is replaced with dT (CP5.47 TTXT). This loss of binding was also observed in ITC experiments (Figure 5I). Contrary to the initial hypothesis that a modification in the DS would promote target‐induced refolding, the nucleobase modification in the DS does not contribute to binding. The double‐modified CP5.47 XTXT binds to kanamycin A with a K_D_ of 8 ± 4 µM, as revealed by ITC (Figure 5J). The binding enthalpy (ΔH) and entropy contribution (‐TΔS) of CP5.47 XTXT remained unchanged after replacing EdU_17_ and EdU_40_ with dT, matching those of CP5.47. Only the stoichiometry increases (Table S5, Supporting Information). Both sites for modifications in CP5.47 XTXT can also be found in CP3 and CP4, suggesting that this binding motif is conserved also in other enriched sequences.

Previous DNA aptamer selections for kanamycin A have employed a range of selection strategies. The Strehlitz group, for example, used a capture SELEX with a DNA SELEX library to identify aptamers binding to kanamycin A [20]. One of the highest‐affinity and most specific sequence, named #3_19, has a reported K_D_ of 3.9 µM, measured using an elution‐based method. Subsequent surface plasmon resonance (SPR) measurements revealed a K_D_ > 300 µM [29]. Although the reported dissociation constant of an aptamer can vary with the characterization method, validation using established techniques such as ITC, SPR, BLI, or microscale thermophoresis (MST) is preferable to ensure a reliable assessment of binding affinity. Another aptamer reported by Song et al., named Ky2, has a reported K_D_ of 79 nM [30]. However, this K_D_ was measured using an immobilized target. The Liu group tried to reproduce the K_D_ using a thioflavin T replacement assay and ITC with kanamycin A in solution, but they were unsuccessful [21]. Either Ky2 has a much higher K_D_ than initially reported, or the aptamer loses its binding affinity for kanamycin A in solution. This discrepancy underscores the importance of selecting and validating aptamers against small molecules in their native, solution‐state form. Additionally, a DNA aptamer for kanamycin A was selected using restriction‐enzyme‐based SELEX (RE‐SELEX) [31]. This aptamer has a reported K_D_ of 340 µM, which is approximately 50 times higher than the K_D_ of CP5.47. Zhao and Liu used pH‐directed capture SELEX to identify a DNA aptamer named Kan6‐1 [21]. The sequence exhibits a K_D_ of 0.3 µM at pH 6.0, but loses affinity at pH 8.0. By lowering the pH, kanamycin A becomes slightly positively charged, which generally facilitates aptamer selection. Unlike CP5, Kan6‐1 exhibits strong cross‐reactivity with tobramycin, indicating lower target specificity. Overall, kanamycin A was used as a target to develop different selection methods, yielding aptamers with distinct characteristics, making it a suitable model target for establishing a new method.

Other nucleobase‐modified aptamers identified by click SELEX that bind to small molecules were described [16, 32]. For example, a boronic acid‐modified aptamer binding to epinephrine was identified [32]. This clickmer binds epinephrine with a K_D_‐value of 1 µM, comparable to the one of CP5 and kanamycin A. The nucleobase modification was introduced through strain‐promoted alkyne‐azide cycloaddition to a dibenzocyclooctyne‐modified dUTP and using a particle display strategy.

In the click capture SELEX, a moderate selection stringency was employed, and high target concentrations were applied during the elution step. As described by Alkhamis et al. [28], the enrichment of sequences binding to flunixin with low nanomolar affinity was accomplished by decreasing the target concentration from the initiation of the selection and augmenting selection stringency with marked sharpness. This more stringent selection condition could also be applied to click capture SELEX, however, within the limits of the occurrence of replication artifacts.

## 3 Conclusion

The results of this study demonstrate the feasibility of using nucleobase‐modified DNA sequences in capture SELEX. Notwithstanding the appearance of by‐products and replication artifacts during the enrichment process, kanamycin A‐binding sequences were identified. A more elaborate analysis was conducted on one sequence (CP5) with respect to specificity, click‐in dependency, and affinity. CP5 has a K_D_ value in the low micromolar range, which is ∼100 times lower than the lowest target concentration used in the selection. Nevertheless, a by‐product that emerged during the selection process and high mutation rates in the DS limit the methodology. These issues can be addressed by employing different library designs, including modified primer‐binding sites [24], docking sequences without nucleobase modifications, or by altering the positioning of the docking sequence within the library [33]. However, the by‐product formation did not impede the enrichment of sequences capable of binding to the target, despite being enriched at low frequencies (1.3% and 0.26%). Consequently, the data demonstrate the capability of aptamer identification at early enrichment stages, even when the library is predominantly composed of non‐binding sequences. Small molecules are chemically diverse in size, shape, and functional groups. This, in conjunction with the limited availability of epitopes, suggests that aptamers with versatile chemical features may be advantageous for achieving stronger interactions. However, there is a paucity of reports on the subject of modified aptamers, including nucleobase‐modified aptamers, XNAs, and artificial nucleobases, that bind to small molecules [16, 32, 34]. All of these modified aptamers were identified using a selection method that relies on target modification, either for bead immobilization [16, 34] or for fluorescence labeling [32]. In contrast, modification of the target molecule is not necessary in click capture SELEX.

The described methodology offers a flexible and versatile SELEX platform for the generation of nucleobase‐modified aptamers. The development of click capture SELEX represents an important advancement in the field, extending the toolkit for identifying aptamers that bind small molecules. The method described herein has the potential for broad applicability to diverse targets, especially in cases where the formation of replication artifacts can be prevented. The approach facilitates the implementation of diverse chemical entities and enables flexible downstream analysis of clickmers. The underlying mechanism of click chemistry allows for expeditious substitution of nucleobase modifications, thereby allowing for the alteration and enhancement of functionality.

## 4 Experimental Section

### 4.1 Material

The azides were purchased from Enamine, except the 3‐(2′‐azidoethyl)‐*1H*‐indole, which was synthesized in our lab. The agarose, dATP,dCTP, dGTP, and dTTP were purchased from GENAXXON Bioscience, the CuSO_4_ from Caesar & Loretz and the ethidium bromide, KH_2_PO_4_, K_2_HPO_4_, NaH_2_PO_4_, MgCl_2_, Tween‐20, gentamicin sulfate and TRIS from Carl Roth. The D‐(+)‐Glucose is purchased from Merck. From Sigma Aldrich, we ordered the DMSO, Na_2_EDTA, kanamycin A sulfate, ribostamycin sulfate, tobramycin, paromomycin and (+)‐sodium L‐ascorbate. Tris(3‐hydroxypropyltriazolylmethyl)amin (THPTA) and E‐dUTP were purchased from BaseClick. From LKT Laboratories we purchased kanamycin B. The λ‐Exonuclease, the Gene Ruler ultra‐low range, Quant‐iT OliGreen ssDNA Reagent, Hexafluor‐2‐Propanol (HFIP), triethylammonium (TEA) and the Dynabeads M‐280 Streptavidin were purchased from Thermo Fisher Scientific. GoTaq DNA Polymerase, 5x GoTaq Colorless Reaction Buffer and 25 mM MgCl_2_ for PCR were purchased from Promega. All DNA sequences were purchased from Ella Biotech or Biomers.

### 4.2 CuAAC

A 100 mM solution of sodium ascorbate was freshly prepared. The catalyst solution was prepared by pipetting 140 µL ddH_2_O, THPTA (4 mM), CuSO_4_ (1 mM), and the sodium ascorbate solution (25 mM) in the specified order. The catalyst solution was incubated for 15 min. In the meantime, the reaction mixture was prepared by mixing the Phosphate buffer (6.15% (vol/vol) 1 M K_2_HPO_4_ and 3.85% (vol/vol) 1 M KH_2_PO_4_ in ddH_2_O at pH 7), the DNA template (maximal 500 pmols) and the desired azide in DMSO (1 mM). The reaction is initiated by adding 10 µL of the catalyst solution to a final volume of 100 µL. The reaction mixture is incubated at 37°C for 60 min. Afterwards, the reaction mixture is purified using a Macherey Nagel Gel and PCR clean up Kit from Macherey Nagel or Amicon Ultra 0.5 mL centrifugal filters from Merck, following the instructions of the manufacturers

### 4.3 Duplex Stability of Modified‐DNA Using Bio‐Layer Interferometry

To evaluate nucleobase modifications on DNA duplex formation and stability, a biotinylated docking sequence was used. The DS was modified with different chemical groups according to the CuAAC protocol. The samples were purified using Amicon Ultra 0.5 mL centrifugal filters from Merck, followed by an HPLC purification. For the HPLC purification, the samples were loaded onto an EC Nucleodur C18 gravity column (4.6×125 mm, 5 µM) from Macherey‐Nagel, and a gradient of 100 mM HFIP with 10 mM TEA (Solvent A) and acetonitrile (Solvent B) with a flow rate of 0.3 mL*min^−1^ as the mobile phase at a column temperature of 25°C was used. The DNA samples were purified with the following linear gradient elution conditions (minutes/B%): 0/2, 5/8, 18/18, 20/25, 22/25, 30/2. The collected samples were incubated in a SpeedVac to evaporate the HPLC solvents. The samples were resuspended in SELEX buffer (SB) (137 mM NaCl, 2.7 mM KCl, 10.1 mM NaH_2_PO4, 1.8 mM K_2_HPO4 and 3 mM MgCl_2_, pH 7.5).

The experiments were performed at 21°C using an Octet R8 BLI instrument from Sartorius and streptavidin SAX probes from Sartorius. The biotinylated targets were prepared at 50 nM in HBS‐EP+ buffer from Cytiva and immobilized onto the SAX probes in this buffer for 4 min. Then, free biotin, prepared in HBS‐EP+, at 2.4 µM was loaded onto the probes to saturate streptavidin. The buffer was changed to SB with 0.05% Tween20. The reverse strands were prepared in this buffer at increasing concentrations up to 1500 nM (dilution factor 4). Contact time for the association and dissociation phases was 60 and 300 s, respectively. The sensorgrams were fitted to a 1:1 interaction model using Octet Analysis Studio software (v13.0.0.32) from Sartorius. The dissociation equilibrium constant, K_D_, was calculated as k_off_/k_on_.

### 4.4 SELEX Procedure

In the first click‐capture SELEX round, 100 pmol of CP‐L was modified via CuAAC. The library was dissolved in the SB and 100 pmol of CapO was added. The mixture was incubated at 50°C with shaking at 600 rpm for 15 min. Afterwards, it was slowly cooled by decreasing the temperature by 2°C every five minutes until reaching 30°C, followed by a final incubation without temperature control for five minutes. Meanwhile, 1 mg of Dynabeads M‐280 Streptavidin was washed twice with 250 µL of binding and wash buffer (10 mM TRIS, 1 mM EDTA, 2000 mM NaCl, pH 7.6) and once with 250 µL of SB. The beads were then resuspended in 50 µL SB. The library‐CapO complex was added and the total volume was adjusted to 150 µL. This solution was incubated at 21°C with shaking at 600 rpm for 60 minutes to facilitate immobilization. Every 15 min, the bead suspension was pipetted up and down to ensure proper mixing. The supernatant was discarded, and the beads were washed ten times with 100 µL SB. Afterwards, the beads were resuspended in 50 µL SB and incubated again at 21°C with shaking at 600 rpm for 60 min to minimize background elution during the selection step. The supernatant was exchanged every 15 minutes. The beads were washed an additional five times with 100 µL SB. Next, 50 µL of kanamycin A (1 mM) dissolved in SB was added to the beads, and the mixture was incubated at 21°C with shaking at 600 rpm for 15 min. The supernatant was removed and 45 µL of it were used as a template for PCR. The PCR mix contained GoTaq Colorless Reaction Buffer (1X), MgCl_2_ (2 mM), Fw Primer (0.5 µM), Rv Primer (0.5 µM), dATP (0.25 mM), dCTP (0.25 mM), dGTP (0.25 mM), EdUTP (0.25 mM), and GoTaq DNA Polymerase (0.05 U/µL), making a final volume of 100 µL with ddH_2_O. The PCR conditions were: 1) 95°C for 300 s; 2) 95°C for 30 s; 3) 62°C for 30 s; 4) 72°C for 60 s; repeated in steps 2–4. The PCR product was analyzed on a 4% agarose gel in TBE buffer containing 0.0001% (vol/vol) Ethidium bromide. When the product was clearly visible, the number of PCR cycles was recorded, and 10 µL of the PCR product were diluted to 100 µL to perform an additional scale‐up PCR with four extra cycles. 70 µL of each scale‐up PCR product were combined and mixed with 7 µL of λ‐Exonuclease. The digestion was carried out at 37°C for 60 min, and the enzyme was inactivated by heating the mixture to 80°C for 10 min. The sample was purified using a Macherey‐Nagel Gel and PCR Clean‐up Kit according to the instructions of the manufacturer. The next SELEX cycle started with library modification via CuAAC. From the second cycle onward, 20 pmol of the library were used. The target concentration was lowered to 0.5 mM beginning in the seventh cycle. The incubation time with the target was reduced to ten minutes in rounds five through eight, and to five minutes in rounds nine through ten.

### 4.5 DNA Sequencing

We prepared each SELEX round, including the starting library for next‐generation sequencing, according to the protocol described by Tolle and Mayer [35].

We amplified each SELEX library using PCR. For each PCR reaction, 1 µL of the SELEX rounds was mixed with PfU reaction buffer (1X), MgCl_2_ (2 mM), Index Fw Primer (1 µM), Index Rv Primer (1 µM), dATP (0.25 mM), dCTP (0.25 mM), dGTP (0.25 mM), dTTP (0.25 mM) and homemade PfU DNA Polymerase (0.05 U/µL). The PCR reaction was filled up to 200 µL with ddH_2_O. Eight PCR cycles were performed according to a PCR program described in the SELEX procedure. The PCR product was purified using a Macherey‐Nagel Gel and PCR Clean‐up Kit according to the manufacturer's instructions. Then, equal amounts of DNA were mixed to a total of 1–2 µg in a final volume of 60 µL. For the subsequent library preparation, a TruSeq DNA PCR‐Free Sample Preparation Kit LT from Illumina was used. First, 40 µL of the End Repair Mix 2 were added to the DNA. The mixture was incubated at 30°C for 30 min, followed by a purification using Macherey‐Nagel Gel and PCR Clean‐up Kit. The sample was eluted in 40 µL resuspension buffer supplied by the TruSeq DNA PCR‐Free Sample Preparation Kit LT. Second, 20 µL of A‐Tailing‐Mix was added for adenylation of the 3’‐ends. The mixture was incubated at 37°C for 30 min, 70°C for 5 min and 4°C for 5 min. Thereafter, 5 µL of Ligation mix 2 and 5 µL of the adapter were added. The mixture was incubated at 30°C for 10 min and at 4°C for 5 min. This step was followed by preparative gel purification, during which only the longest sequence with ligated adapters at both sites was cut out. The DNA was purified using the Macherey–Nagel Gel and PCR Clean‐up Kit. The DNA was eluted in the resuspension buffer. Illumina sequencing was performed with 75 bp single‐end sequencing. The sequencing was conducted by the members of the PRECISE platform from DZNE in Bonn, Germany. The analysis of the NGS data was performed using in‐house‐designed software.

### 4.6 Mutational Analysis of the Docking Sequence

The docking sequence was identified using a self‐written Python (v. 3.10.16) script that compared the docking sequence (DS) with sequences from the NGS data. For sequences with a mutated DS, the part with the most frequent matches was used. The starting position of the annotated DS and the number of base mismatches were determined. To simplify our dataset, only sequences with fewer than six mutations and a frameshift of fewer than six nucleotides were selected for further data processing.

### 4.7 Quantification of Sequences Immobilized on Magnetic Beads

20 pmol of a nucleobase‐modified SELEX library were immobilized on Dynabeads *M*‐280 Streptavidin (SA‐beads) according to the protocol described in the SELEX procedure. After the immobilization, the SA‐beads were washed three times with 100 µL SB. After washing, the beads were resuspended in 50 µL SB and incubated at 80°C and 900 rpm for 15 min to elute the sequences. The supernatant was collected and diluted 1:50 in SB. 50 µL of the solution were mixed with 50 µL of an Oligreen solution (TE‐buffer, Quant‐iT OliGreen ssDNA Reagent) and the whole volume was transferred to a Corning 96 Well Half‐Area Microplate from Sigma Aldrich. After ten minutes of incubation in the dark, the fluorescence intensity was measured using a Spark Multimode Microplate Reader from Tecan at an excitation wavelength of 485 nm and emission wavelength of 535 nm. For the CP‐library samples, both modified and unmodified, we had to dilute them 1:5 because the fluorescence intensity exceeded the instrument's range. We accounted for dilution during data evaluation by multiplying the fluorescence intensity by 5. The fluorescence intensities were normalized using Min‐Max normalization.

### 4.8 Fluorescence Polarization‐Based Binding Assay

We adapted the fluorescence polarization assay from Legen and Mayer [26].

The assay was performed in a final volume of 20 µL SB (recipe described in the SELEX procedure) with 0.05% Tween20. First, the DNA (500 nM) and the indicated concentration of small‐molecule targets were mixed and incubated at 21°C and 500 rpm for 30 min. As a control, a sample without DNA was incubated for each target concentration. Second, Cy3‐labeled capture oligonucleotide (Cy3‐ODN) was added. For the libraries and the non‐truncated monoclonal sequences, Cy3‐ODN was added at 0.1 nM. For CP5.47, Cy3‐ODN was added at 0.5 nM. The sample was incubated at 21°C and 500 rpm for another 30 minutes. Finally, the sample was transferred to a Corning 384 Well Microplate from Sigma Aldrich. The fluorescence polarization at excitation light 535 nm and emission light 595 nm was measured by Tecan Ultra Micro Plate Reader from Tecan.

In all experiments analyzing Cy3‐ODN‐DNA interactions, the first step was omitted, and the Cy3‐ODN was added directly to the solution.

### 4.9 Binding Assay Using ITC

The clickmer samples used for affinity measurements were modified as described in CuAAC. After purifying the samples using Amicon Ultra 0.5 mL centrifugal filters from Merck, we evaporated the solvent using the Concentrator Plus from Eppendorf. In the meantime, SB was prepared and degassed for five minutes by ultrasonication and vacuum. After that, the DNA sample and kanamycin A (100 µM) were dissolved in SB. Both, the target and the clickmer, were centrifuged at 21130 rcf for five minutes. Kanamycin A in the syringe was titrated to different clickmer concentrations in the cell. For the affinity measurements, a MicroCal PEAQ‐ITC from Malvern Panalytical was used. 19 injections of kanamycin A were titrated to the clickmer in the cell: first injection 0.4 µL followed by 18 injections of 2 µL. The measurements were performed at 21°C. For the CP5.47 XTXT, the syringe was refilled again with kanamycin A without cleaning the sample cell, and the titration was repeated to reach saturation. The integrated binding isotherm was fitted to a one set of sites binding model using the MicroCal PeaQ ITC Analysis Software (v 1.41).

### 4.10 Data Analyzation

Unless otherwise specified, the data analysis was performed in Python (v. 3.10.16). Data handling and preprocessing were carried out using pandas (v. 2.3.3) and NumPy (v. 2.2.4). The figures were generated with Seaborn (v. 0.13.2) and Matplotlib (v. 3.10.1). Statistical analysis was conducted using SciPy (v. 1.15.3) and statsmodels (0.14.4). The assumption of normality was evaluated using the Kolmogorov‐Smirnov test. Depending on the experimental design, statistical significance was assessed using t‐tests or ONE‐way ANOVA, followed by Tukey's post hoc test. Significance levels were reported as *p*< 0.05 = ‘*’, *p* < 0.01 = ‘**’, and *p*< 0.001 = ‘***’.

## Conflicts of Interest

The authors declare no conflicts of interest.

The data that support the findings of this study are available from the corresponding author upon reasonable request.

## Supporting information



The authors have cited no additional references within the Supporting Information.
