# Supplementary material for: Click Capture SELEX for the Identification of Click‐Modified Aptamers Targeting Small Molecules in Solution
Source: Chemistry. 2026 Apr 4;32(23):e70932. doi: 10.1002/chem.70932 (PMC13282910; doi:10.1002/chem.70932)
Supplement: Supplementary file 1 — The authors have cited no additional references within the Supporting Information. [file CHEM-32-e70932-s001.docx]

**Table S1:** DNA sequences used in this study

| Name | Sequence |
| --- | --- |
| CP-library (CP-L) | 5‘-CACGACGCAAGGGACCACAGG-N_10_-AGAGGC(EdU)CGAAC-N_25_-AGCACGACACCGCAGAGGCA-3‘  (dA:dC:dG:EdU 1:1:1:0.35) |
| CP-L(dA).12 | 5‘-CACGACGCAAGGGACCACAGG-N_10_-AAAAAAAAAAAA-N_25_-AGCACGACACCGCAGAGGCA-3‘  (dA:dC:dG:EdU 1:1:1:0.35) |
| Forward Primer | 5‘-CACGACGCAAGGGACCACAGG-3‘ |
| Reverse Primer | 5‘-Phosphate-TGCCTCTGCGGTGTCGTGCT-3‘ |
| NGS Fw Primer SL | 5‘-GGCTACACGACGCAAGGGACCACAGG-3‘ |
| NGS Fw Primer R1 | 5‘-ATCACGACGACGCAAGGGACCACAGG-3‘ |
| NGS Fw Primer R2 | 5‘-CGATGTACGACGCAAGGGACCACAGG-3‘ |
| NGS Fw Primer R3 | 5‘-TTAGGCACGACGCAAGGGACCACAGG-3‘ |
| NGS Fw Primer R4 | 5‘-TGACCAACGACGCAAGGGACCACAGG-3‘ |
| NGS Fw Primer R5 | 5‘-ACAGTGACGACGCAAGGGACCACAGG-3‘ |
| NGS Fw Primer R6 | 5‘-GCCAATACGACGCAAGGGACCACAGG-3‘ |
| NGS Fw Primer R7 | 5‘-CAGATCACGACGCAAGGGACCACAGG-3‘ |
| NGS Fw Primer R8 | 5‘-ACTTGAACGACGCAAGGGACCACAGG-3‘ |
| NGS Fw Primer R9 | 5‘-GATCAGACGACGCAAGGGACCACAGG-3‘ |
| NGS Fw Primer R10 | 5‘-TAGCTTACGACGCAAGGGACCACAGG-3‘ |
| NGS Rv Primer SL | 5‘-GGCTACTGCCTCTGCGGTGTCGTGCT-3‘ |
| NGS Rv Primer R1 | 5‘-ATCACGTGCCTCTGCGGTGTCGTGCT-3‘ |
| NGS Rv Primer R2 | 5‘-CGATGTTGCCTCTGCGGTGTCGTGCT-3‘ |
| NGS Rv Primer R3 | 5‘-TTAGGCTGCCTCTGCGGTGTCGTGCT-3‘ |
| NGS Rv Primer R4 | 5‘-TGACCATGCCTCTGCGGTGTCGTGCT-3‘ |
| NGS Rv Primer R5 | 5‘-ACAGTGTGCCTCTGCGGTGTCGTGCT-3‘ |
| NGS Rv Primer R6 | 5‘-GCCAATTGCCTCTGCGGTGTCGTGCT-3‘ |
| NGS Rv Primer R7 | 5‘-CAGATCTGCCTCTGCGGTGTCGTGCT-3‘ |
| NGS Rv Primer R8 | 5‘-ACTTGATGCCTCTGCGGTGTCGTGCT-3‘ |
| NGS Rv Primer R9 | 5‘-GATCAGTGCCTCTGCGGTGTCGTGCT-3‘ |
| NGS Rv Primer R10 | 5‘-TAGCTTTGCCTCTGCGGTGTCGTGCT-3‘ |
| Capture Oligo | 5‘-Biotin-HEG-GTTCGAGCCTCT-3‘ |
| Cy3 Capture Oligo (Cy3 ODN) | 5‘-Cy3-GTTCGAGCC-3‘ |
| CP1 | 5‘-CACGACGCAAGGGACCACAGGGGA(EdU)GC(EdU)GGAAGAGGC(EdU)CGAACGAG(EdU)CCAGAAGGAACAAAGGAGGAAAGCACGACACCGCAGAGGCA-3‘ |
| CP3 | 5‘-CACGACGCAAGGGACCACAGGGGGA(EdU)GGCGCAGAGGC(EdU)CGAACCACAAGCAAGGAAC(EdU)GGCAAGGGAAAGCACGACACCGCAGAGGCA-3‘ |
| CP4 | 5‘-CACGACGCAAGGGACCACAGGGGA(EdU)GGCGCAGAGGC(EdU)CGAACCACAAGCAAGGAAC(EdU)GGCAAGGGAAAAGCACGACACCGCAGAGGCA-3‘ |
| CP5 | 5‘-CACGACGCAAGGGACCACAGGACGG(EdU)ACGGGAGAGGC(EdU)CGAACAGGAAGAACCGCAC(EdU)CC(EdU)ACCGGAAAGCACGACACCGCAGAGGCA-3‘ |
| CP5 scr | 5‘-CACGACGCAAGGGACCACAGGGGGCG(EdU)AAGCAGAGGC(EdU)CGAACAGAGCACAACCGCCAGGCC(EdU)GAA(EdU)AAGCACGACACCGCAGAGGCA-3‘ |
| CP5.47 | 5‘-ACGG(EdU)ACGGGAGAGGC(EdU)CGAACAGGAAGAACCGCAC(EdU)CC(EdU)ACCGGAA-3‘ |
| CP5.47 scr | 5‘-GGGCG(EdU)AAGCAGAGGC(EdU)CGAACAGAGCACAACCGCCAGGCC(EdU)GAA(EdU)A-3‘ |
| CP6 | 5‘-CACGACGCAAGGGACCACAGGAAA(EdU)GAGGAAAGAGGC(EdU)CGAACGGGGC(EdU)ACCACCAA  AAAGGGG(EdU)AGAAGCACGACACCGCAGAGGCA-3‘ |
| CP5.47 TXXX | 5‘-ACGGTACGGGAGAGGC(EdU)CGAACAGGAAGAACCGCAC(EdU)CC(EdU)ACCGGAA-3‘ |
| CP5.47 XTXX | 5‘-ACGG(EdU)ACGGGAGAGGCTCGAACAGGAAGAACCGCAC(EdU)CC(EdU)ACCGGAA-3‘ |
| CP5.47 XXTX | 5‘-ACGG(EdU)ACGGGAGAGGC(EdU)CGAACAGGAAGAACCGCACTCC(EdU)ACCGGAA-3‘ |
| CP5.47 XXXT | 5‘-ACGG(EdU)ACGGGAGAGGC(EdU)CGAACAGGAAGAACCGCAC(EdU)CCTACCGGAA-3‘ |
| CP5.47 TTXT | 5‘-ACGGTACGGGAGAGGCTCGAACAGGAAGAACCGCAC(EdU)CCTACCGGAA-3‘ |
| CP5.47 XTXT | 5‘-ACGG(EdU)ACGGGAGAGGCTCGAACAGGAAGAACCGCAC(EdU)CCTACCGGAA-3‘ |
| CP-DS | 5‘-Biotin-TEG-AGAGGC(EdU)CGAAC-3‘ |
| CP-DS-dT | 5‘-Biotin-TEG-AGAGGCTCGAAC-3‘ |
| CP-DS-Rev | 5‘-GTTCGAGCCTCT-3‘ |

A) B)

C) D)

E) F)

G) H)

**Figure S1**: BLI sensorgrams to evaluate the binding kinetics of CP-DS_dT (A), unmodified CP-DS (B), and CP-DS modified with EA-dU (C), Imi-dU (D), Tol-dU (E), Phe-dU (F), Ind-dU (G) and Nap-dU (H) to CP-DS-Rev. CP-DS-Rev was added to the immobilized CP-DS at concentrations of 1500 nM, 375 nM, 93.8 nM and 23.4 nM (from the top to the bottom). Thin red lines represent fits of the experiment curves to a 1:1 model interaction.


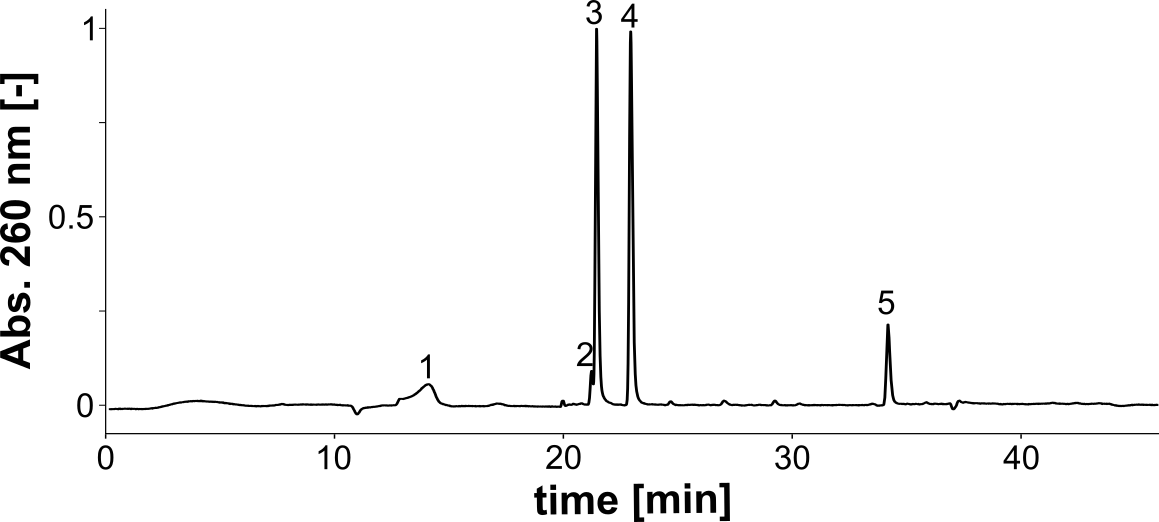


| Peak | Assignments | Molecule | Calc. Mass | Measured Mass |
| --- | --- | --- | --- | --- |
| 1 | dC | [M + H]^+^ | 228.22 | 228.20 |
| 2 | EA-dU | [M + H]^+^ | 339.33 | 339.31 |
| 3 | dA | [M + H]^+^ | 252.25 | 252.25 |
| 4 | dG | [M + H]^+^ | 268.25 | 268.24 |
| 5 | Biotin(TEG)-dA | [M + H]^+^ | 821.85 | 821.84 |

**Figure S2:** QC LC/MS CP-DS EA-dU


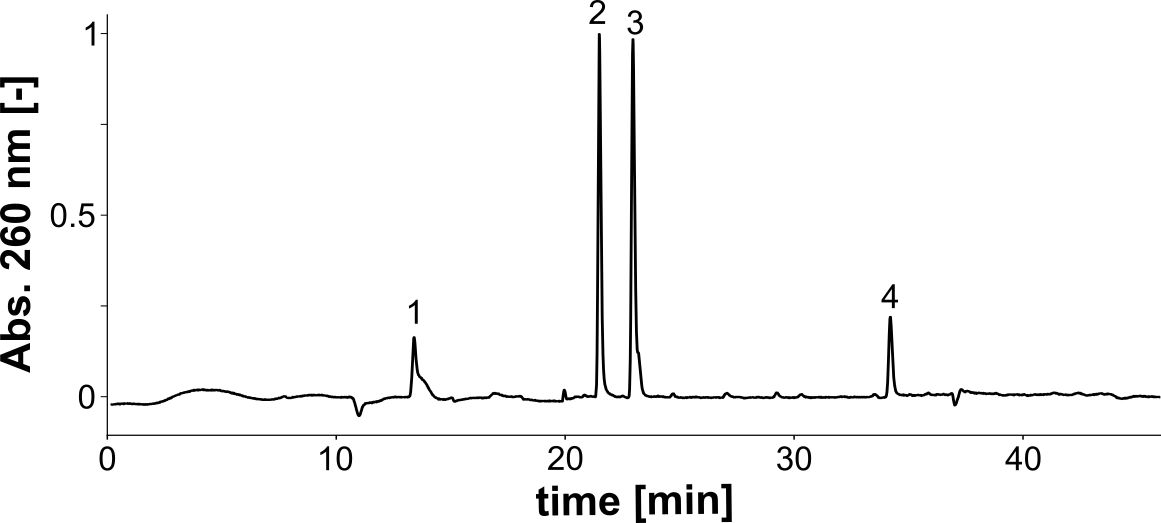


| Peak | Assignments | Molecule | Calc. Mass | Measured Mass |
| --- | --- | --- | --- | --- |
| 1 | dC | [M + H]^+^ | 228.22 | 228.23 |
| 2 | dA | [M + H]^+^ | 252.25 | 252.25 |
| 3 | dG  Imi-dU | [M + H]^+^  [M + H]^+^ | 268.25  390.37 | 268.24  390.33 |
| 4 | Biotin(TEG)-dA | [M + H]^+^ | 821.85 | 821.85 |

**Figure S3:** QC LC/MS CP-DS Imi-dU


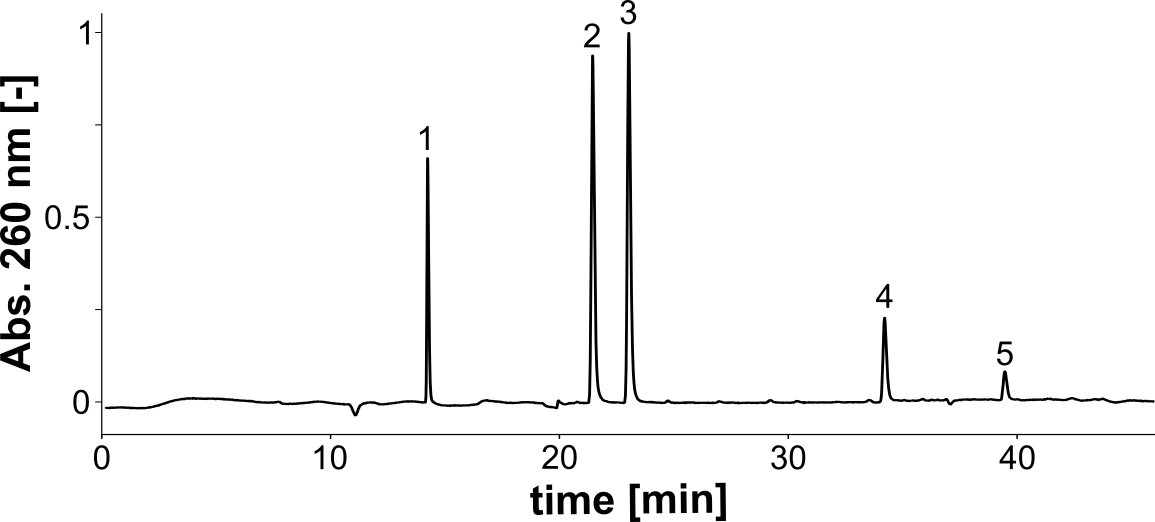


| Peak | Assignments | Molecule | Calc. Mass | Measured Mass |
| --- | --- | --- | --- | --- |
| 1 | dC | [M + H]^+^ | 228.22 | 228.21 |
| 2 | dA | [M + H]^+^ | 252.25 | 252.25 |
| 3 | dG | [M + H]^+^ | 268.25 | 268.25 |
| 4 | Biotin(TEG)-dA | [M + H]^+^ | 821.85 | 821.88 |
| 5 | Tol-dU | [M + H]^+^ | 400.41 | 400.41 |

**Figure S4:** QC LC/MS CP-DS Tol-dU


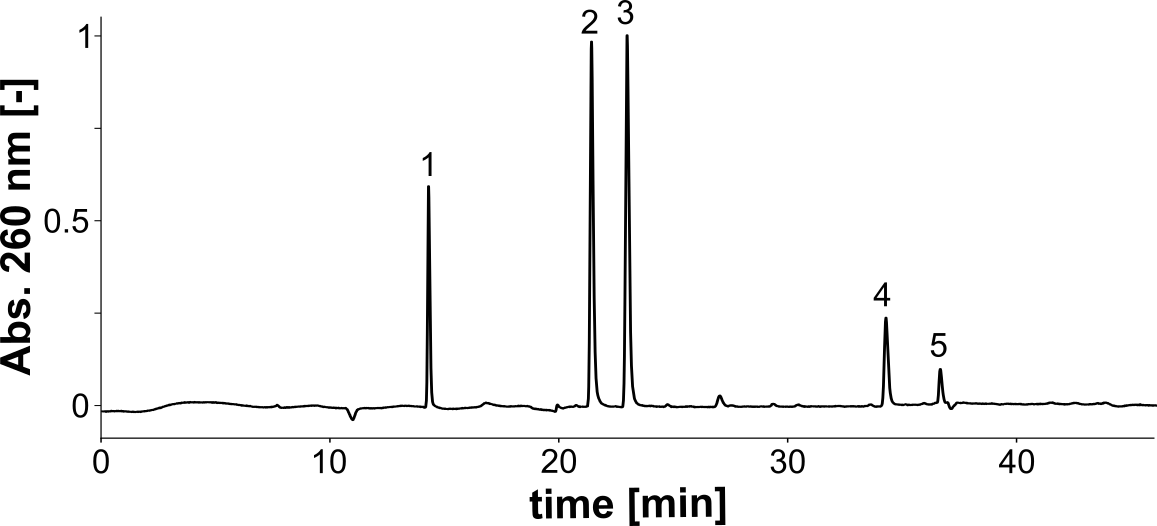


| Peak | Assignments | Molecule | Calc. Mass | Measured Mass |
| --- | --- | --- | --- | --- |
| 1 | dC | [M + H]^+^ | 228.22 | 228.22 |
| 2 | dA | [M + H]^+^ | 252.25 | 252.25 |
| 3 | dG | [M + H]^+^ | 268.25 | 268.25 |
| 4 | Biotin(TEG)-dA | [M + H]^+^ | 821.85 | 821.86 |
| 5 | Phe-dU | [M + H]^+^ | 416.41 | 400.40 |

**Figure S5:** QC LC/MS CP-DS Phe-dU


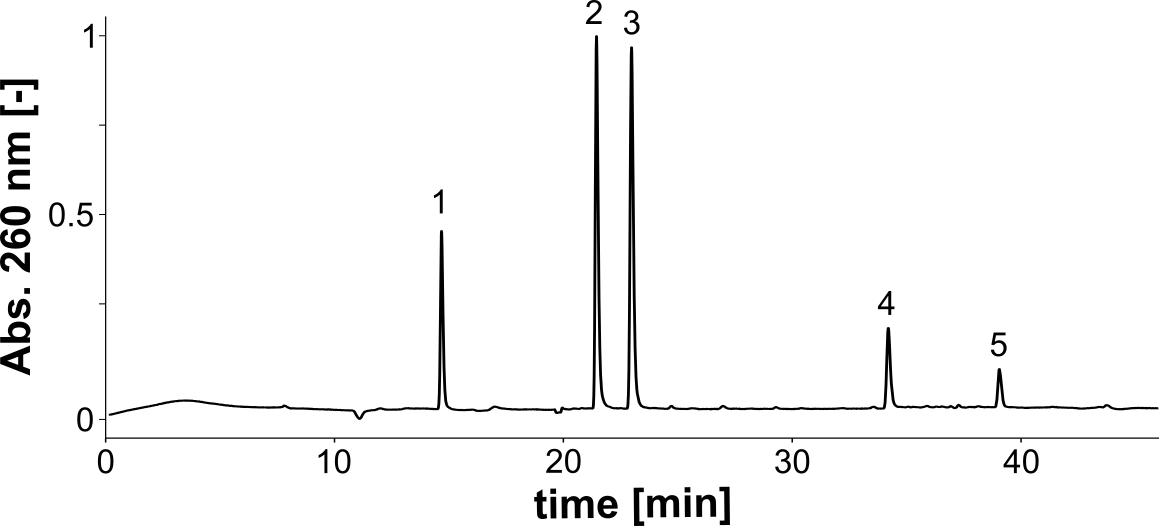


| Peak | Assignments | Molecule | Calc. Mass | Measured Mass |
| --- | --- | --- | --- | --- |
| 1 | dC | [M + H]^+^ | 228.22 | 228.20 |
| 2 | dA | [M + H]^+^ | 252.25 | 252.25 |
| 3 | dG | [M + H]^+^ | 268.25 | 268.25 |
| 4 | Biotin(TEG)-dA | [M + H]^+^ | 821.85 | 821.86 |
| 5 | Ind-dU | [M + H]^+^ | 439.44 | 439.44 |

**Figure S6:** QC LC/MS CP-DS Ind-dU


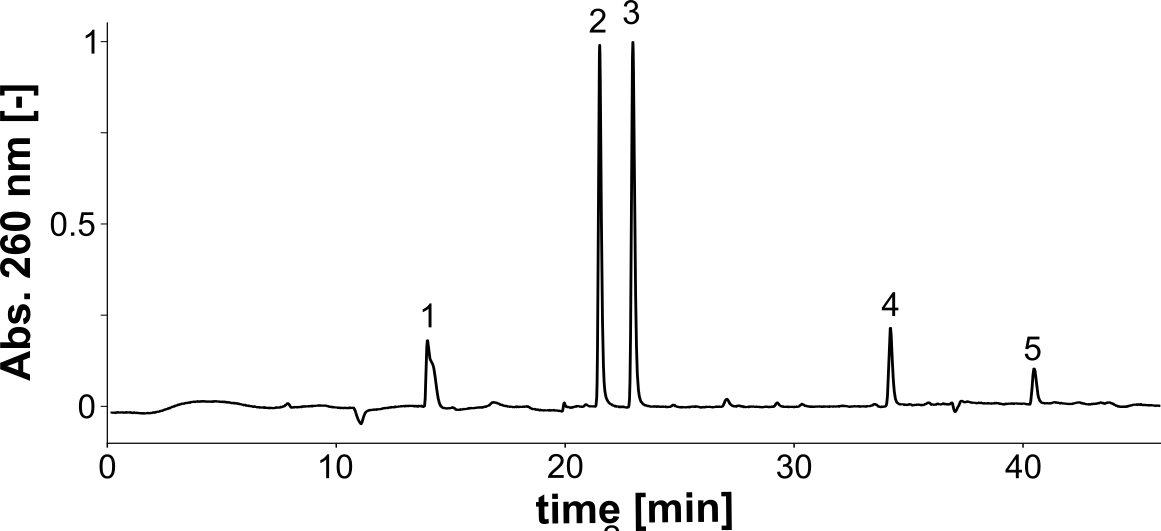


| Peak | Assignments | Molecule | Calc. Mass | Measured Mass |
| --- | --- | --- | --- | --- |
| 1 | dC | [M + H]^+^ | 228.22 | 228.21 |
| 2 | dA | [M + H]^+^ | 252.25 | 252.25 |
| 3 | dG | [M + H]^+^ | 268.25 | 268.24 |
| 4 | Biotin(TEG)-dA | [M + H]^+^ | 821.85 | 821.86 |
| 5 | Nap-dU | [M + H]^+^ | 436.44 | 436.41 |

**Figure S7:** QC LC/MS CP-DS Nap-dU


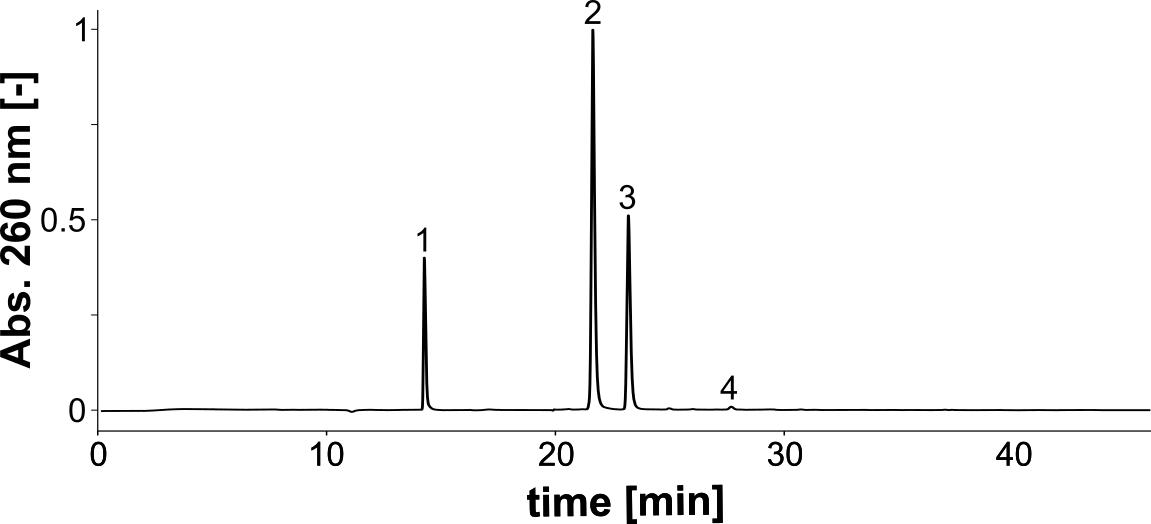


| Peak | Assignments | Molecule | Calc. Mass | Measured Mass |
| --- | --- | --- | --- | --- |
| 1 | dC | [M + H]^+^ | 228.22 | 228.23 |
| 2 | EA-dU  dA | [M + H]^+^ | 339.33  252.25 | 339.32  252.25 |
| 3 | dG | [M + H]^+^ | 268.25 | 268.25 |
| 4 | K-dU | [M + Na]^+^ | 293.23 | 293.23 |

**Figure S8:** QC LC/MS CP-L(dA).12 EA-dU


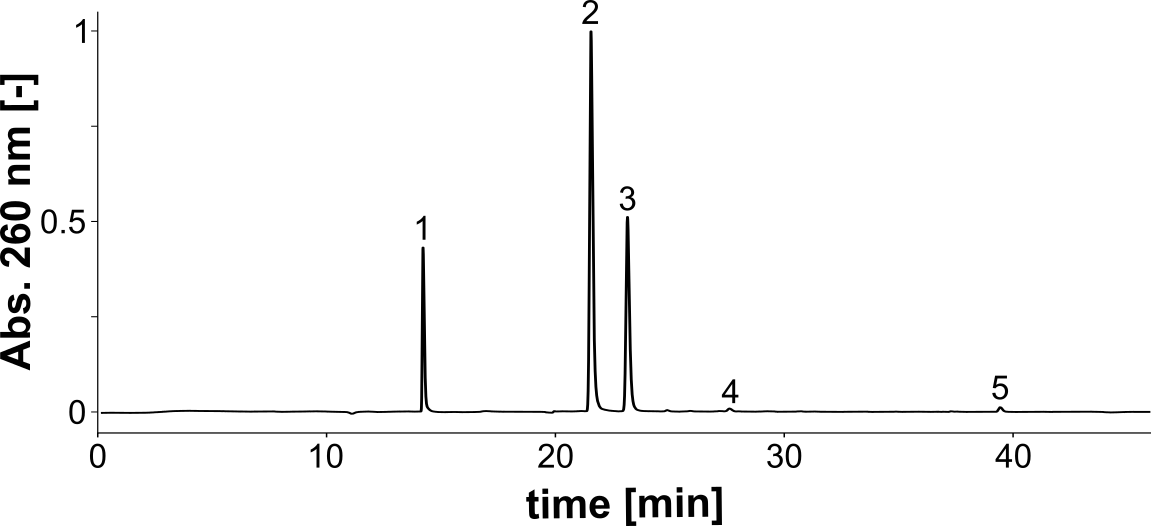


| Peak | Assignments | Molecule | Calc. Mass | Measured Mass |
| --- | --- | --- | --- | --- |
| 1 | dC | [M + H]^+^ | 228.22 | 228.22 |
| 2 | dA | [M + H]^+^ | 252.25 | 252.25 |
| 3 | dG | [M + H]^+^ | 268.25 | 268.24 |
| 4 | K-dU | [M + Na]^+^ | 293.23 | 293.23 |
| 5 | Tol-dU | [M + H]^+^ | 400.41 | 400.41 |

**Figure S9:** QC LC/MS CP-L(dA).12 Tol-dU


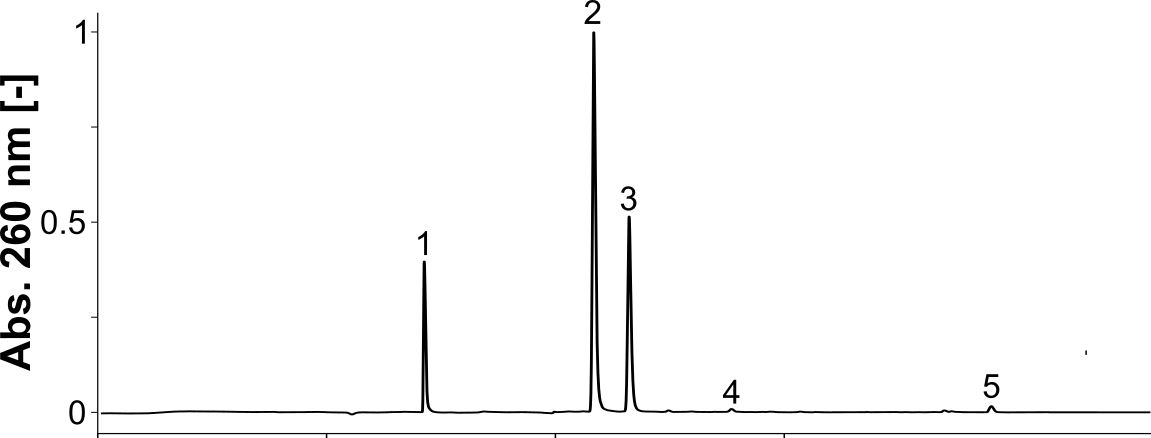


| Peak | Assignments | Molecule | Calc. Mass | Measured Mass |
| --- | --- | --- | --- | --- |
| 1 | dC | [M + H]^+^ | 228.22 | 228.22 |
| 2 | dA | [M + H]^+^ | 252.25 | 252.25 |
| 3 | dG | [M + H]^+^ | 268.25 | 268.24 |
| 4 | K-dU | [M + Na]^+^ | 293.23 | 293.24 |
| 5 | Ind-dU | [M + H]^+^ | 439.44 | 439.45 |

**Figure S10:** QC LC/MS CP-L(dA).12 Ind-dU


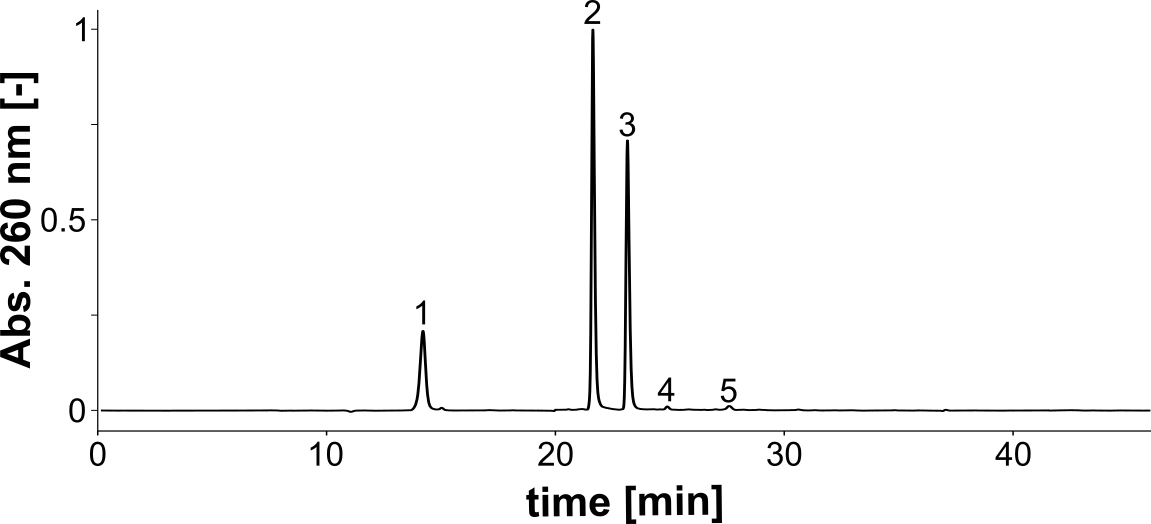


| Peak | Assignments | Molecule | Calc. Mass | Measured Mass |
| --- | --- | --- | --- | --- |
| 1 | dC | [M + H]^+^ | 228.22 | 228.23 |
| 2 | EA-dU  dA | [M + H]^+^ | 339.33  252.25 | 339.32  252.25 |
| 3 | dG | [M + H]^+^ | 268.25 | 268.24 |
| 4 | 8-oxo-dG | [M + H]^+^ | 284.25 | 284.21 |
| 5 | K-dU | [M + Na]^+^ | 293.23 | 293.17 |

**Figure S11:** QC LC/MS CP-L EA-dU


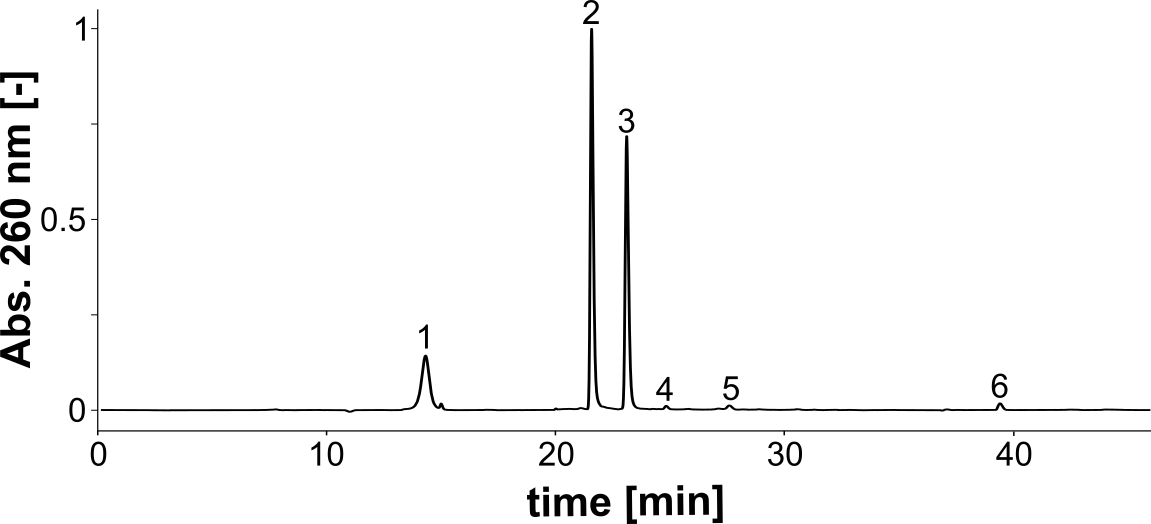


| Peak | Assignments | Molecule | Calc. Mass | Measured Mass |
| --- | --- | --- | --- | --- |
| 1 | dC | [M + H]^+^ | 228.22 | 228.22 |
| 2 | dA | [M + H]^+^ | 252.25 | 252.25 |
| 3 | dG | [M + H]^+^ | 268.25 | 268.25 |
| 4 | 8-oxo-dG | [M + H]^+^ | 284.25 | 284.24 |
| 5 | K-dU | [M + Na]^+^ | 293.23 | 293.18 |
| 6 | Tol-dU | [M + H]^+^ | 400.41 | 400.41 |

**Figure S12:** QC LC/MS CP-L Tol-dU


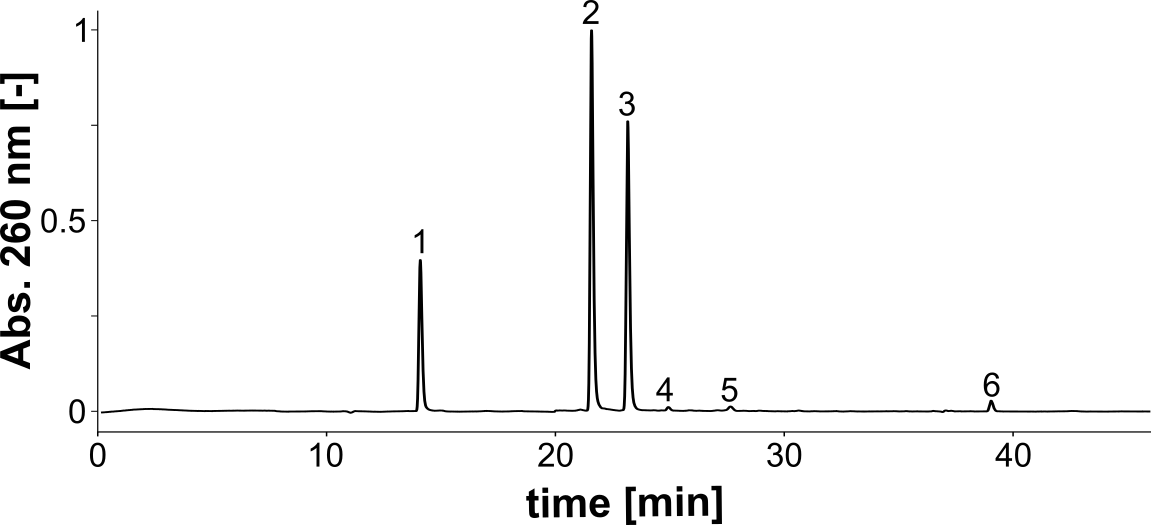


| Peak | Assignments | Molecule | Calc. Mass | Measured Mass |
| --- | --- | --- | --- | --- |
| 1 | dC | [M + H]^+^ | 228.22 | 228.22 |
| 2 | dA | [M + H]^+^ | 252.25 | 252.24 |
| 3 | dG | [M + H]^+^ | 268.25 | 268.23 |
| 4 | 8-oxo-dG | [M + H]^+^ | 284.25 | 284.21 |
| 5 | K-dU | [M + Na]^+^ | 293.23 | 293.18 |
| 6 | Ind-dU | [M + H]^+^ | 439.44 | 439.44 |

**Figure S13:** QC LC/MS CP-L Ind-dU


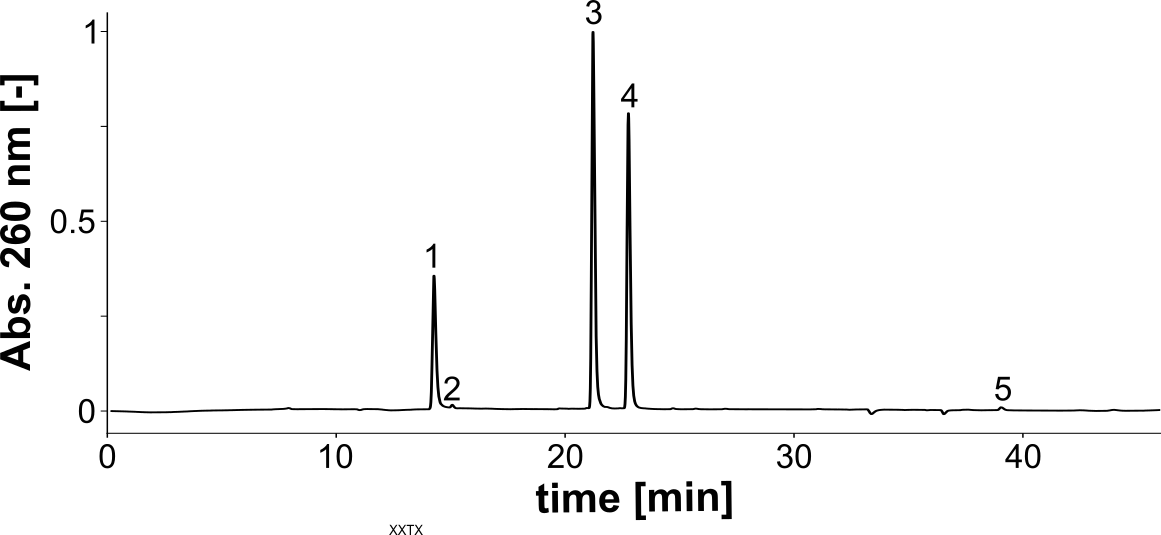


| Peak | Assignments | Molecule | Calc. Mass | Measured Mass |
| --- | --- | --- | --- | --- |
| 1 | dC | [M + H]^+^ | 228.22 | 228.22 |
| 2 | dC | [M + H]^+^ | 228.22 | 228.22 |
| 3 | dA | [M + H]^+^ | 252.25 | 252.25 |
| 4 | dG | [M + H]^+^ | 268.25 | 268.25 |
| 5 | Ind-dU | [M + H]^+^ | 439.44 | 439.45 |

**Figure S14:** QC LC/MS R10 Ind-dU


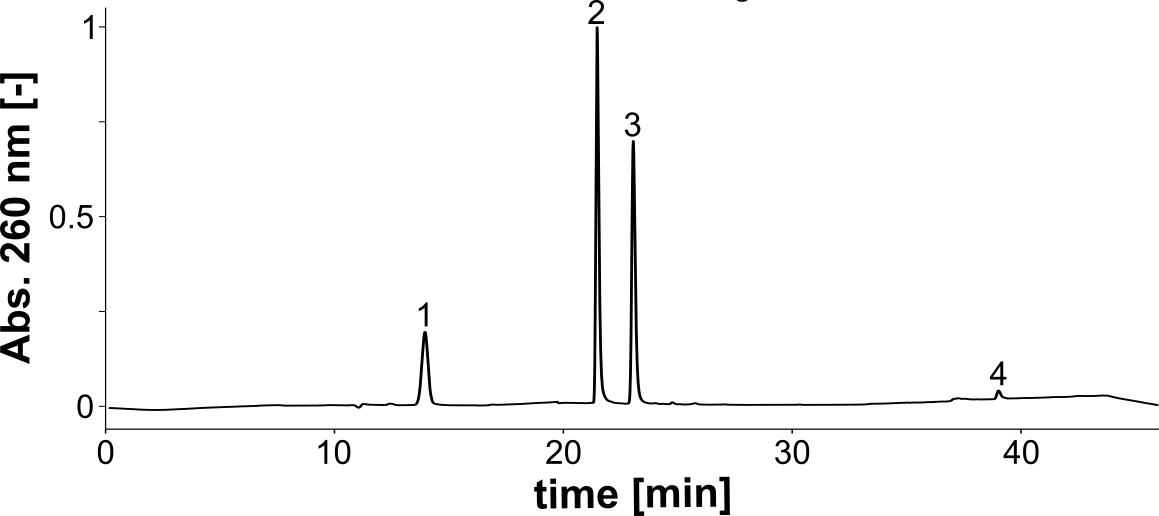


| Peak | Assignments | Molecule | Calc. Mass | Measured Mass |
| --- | --- | --- | --- | --- |
| 1 | dC | [M + H]^+^ | 228.22 | 228.25 |
| 2 | dA | [M + H]^+^ | 252.25 | 252.28 |
| 3 | dG | [M + H]^+^ | 268.25 | 268.28 |
| 4 | Ind-dU | [M + H]^+^ | 439.44 | 439.50 |

**Figure S15:** QC LC/MS CP1 Ind-dU


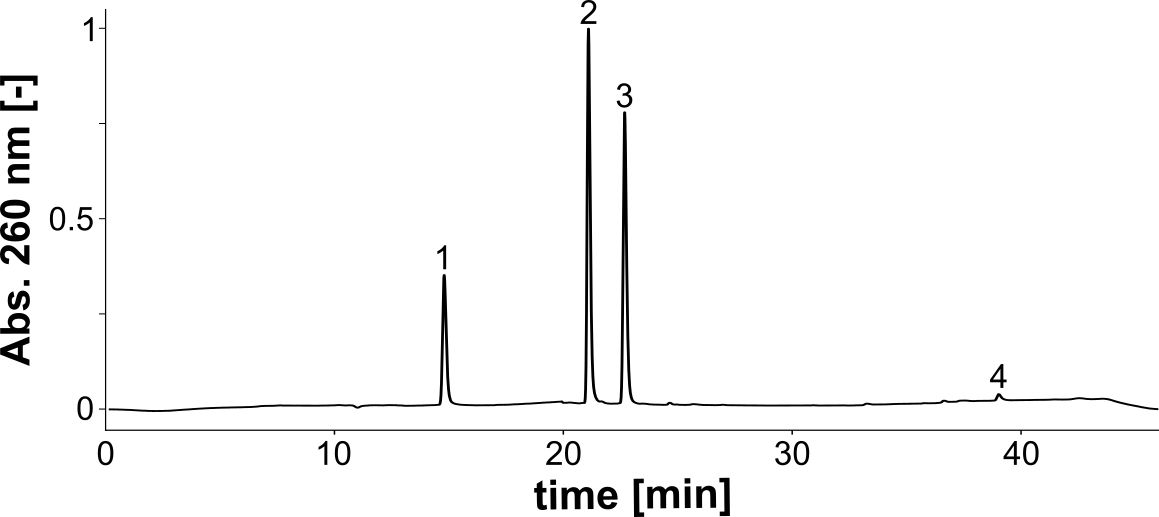


| Peak | Assignments | Molecule | Calc. Mass | Measured Mass |
| --- | --- | --- | --- | --- |
| 1 | dC | [M + H]^+^ | 228.22 | 228.22 |
| 2 | dA | [M + H]^+^ | 252.25 | 252.25 |
| 3 | dG | [M + H]^+^ | 268.25 | 268.24 |
| 4 | Ind-dU | [M + H]^+^ | 439.44 | 439.44 |

**Figure S16:** QC LC/MS CP3 Ind-dU


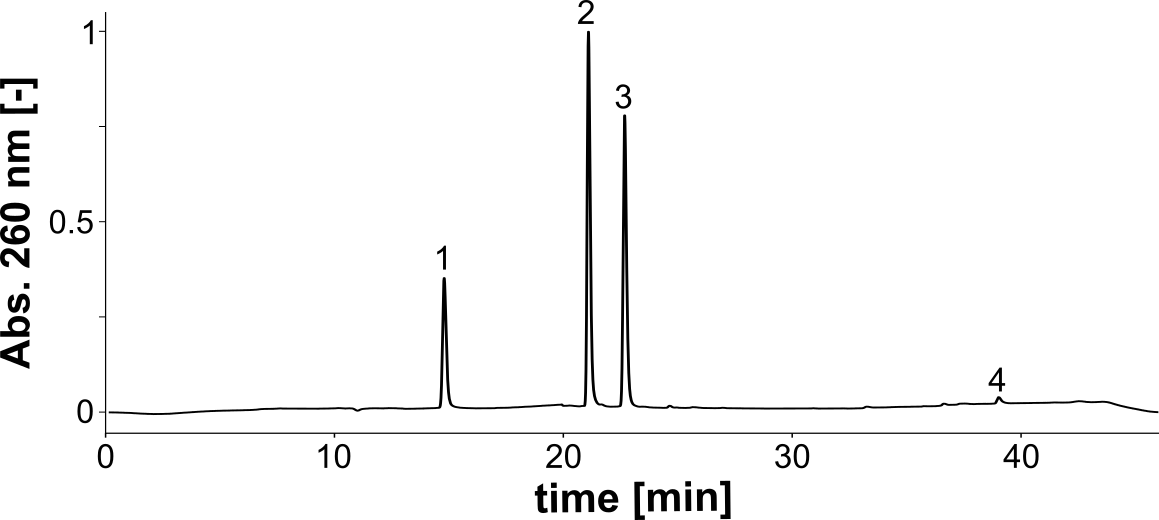


| Peak | Assignments | Molecule | Calc. Mass | Measured Mass |
| --- | --- | --- | --- | --- |
| 1 | dC | [M + H]^+^ | 228.22 | 228.25 |
| 2 | dA | [M + H]^+^ | 252.25 | 252.28 |
| 3 | dG | [M + H]^+^ | 268.25 | 268.28 |
| 4 | Ind-dU | [M + H]^+^ | 439.44 | 439.50 |

**Figure S17:** QC LC/MS CP4 Ind-dU


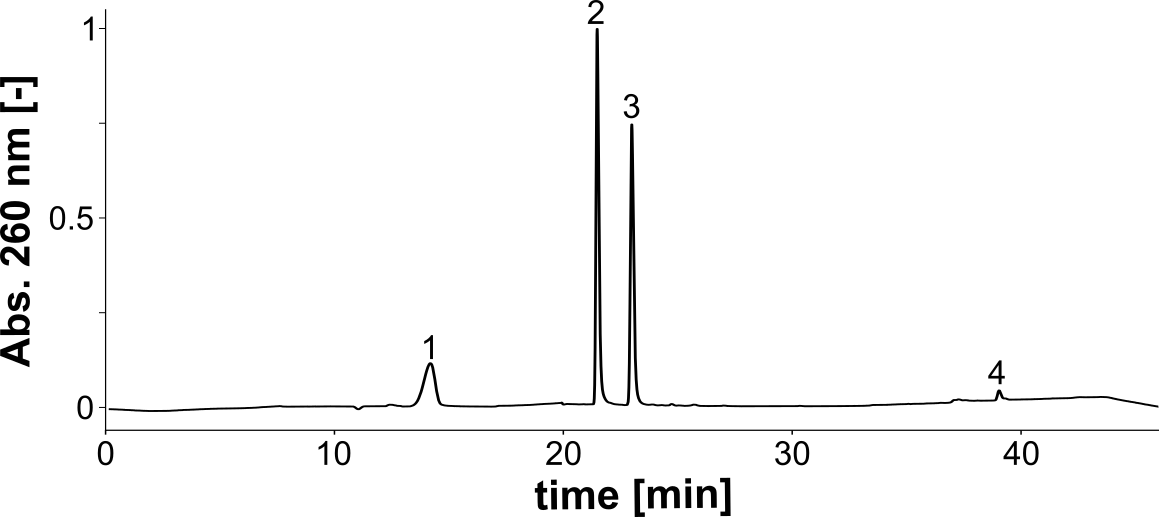


| Peak | Assignments | Molecule | Calc. Mass | Measured Mass |
| --- | --- | --- | --- | --- |
| 1 | dC | [M + H]^+^ | 228.22 | 228.21 |
| 2 | dA | [M + H]^+^ | 252.25 | 252.25 |
| 3 | dG | [M + H]^+^ | 268.25 | 268.23 |
| 4 | Ind-dU | [M + H]^+^ | 439.44 | 439.43 |

**Figure S18:** QC LC/MS CP5 Ind-dU


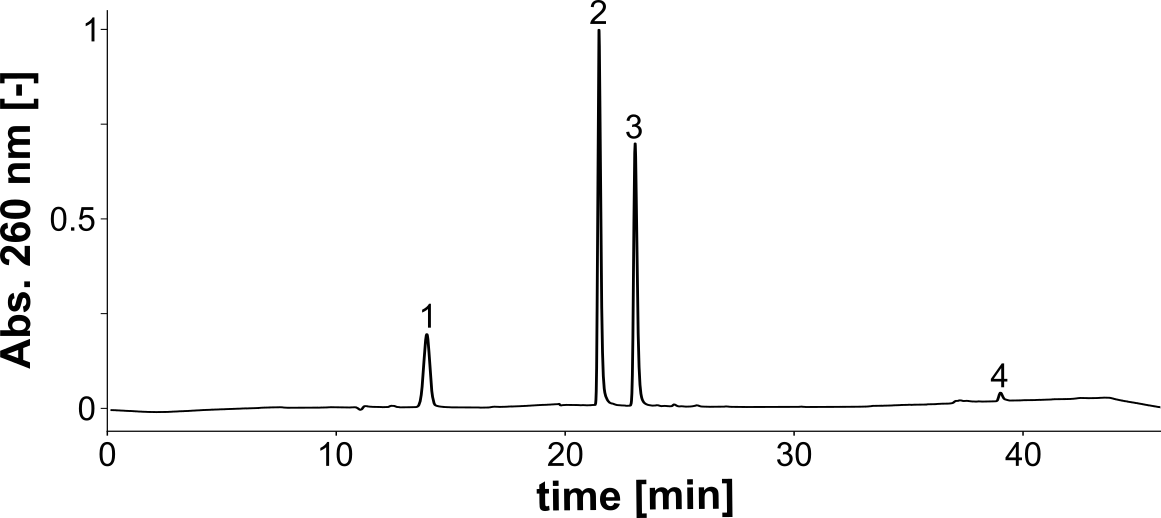


| Peak | Assignments | Molecule | Calc. Mass | Measured Mass |
| --- | --- | --- | --- | --- |
| 1 | dC | [M + H]^+^ | 228.22 | 228.22 |
| 2 | dA | [M + H]^+^ | 252.25 | 252.25 |
| 3 | dG | [M + H]^+^ | 268.25 | 268.24 |
| 4 | Ind-dU | [M + H]^+^ | 439.44 | 439.44 |

**Figure S19:** QC LC/MS CP6 Ind-dU


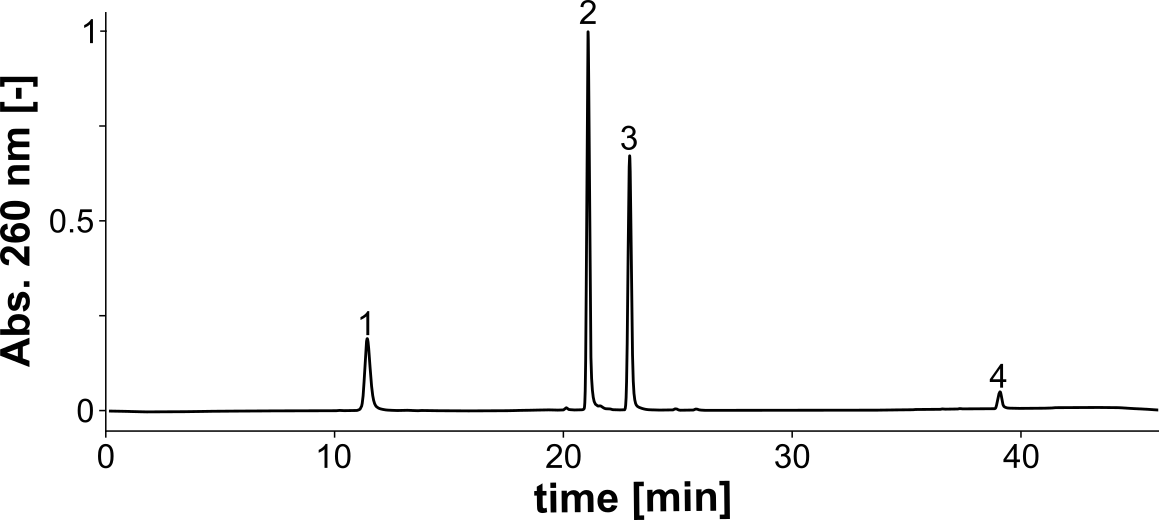


| Peak | Assignments | Molecule | Calc. Mass | Measured Mass |
| --- | --- | --- | --- | --- |
| 1 | dC | [M + H]^+^ | 228.22 | 228.23 |
| 2 | dA | [M + H]^+^ | 252.25 | 252.26 |
| 3 | dG | [M + H]^+^ | 268.25 | 268.25 |
| 4 | Ind-dU | [M + H]^+^ | 439.44 | 439.45 |

**Figure S20:** QC LC/MS CP5 scr Ind-dU


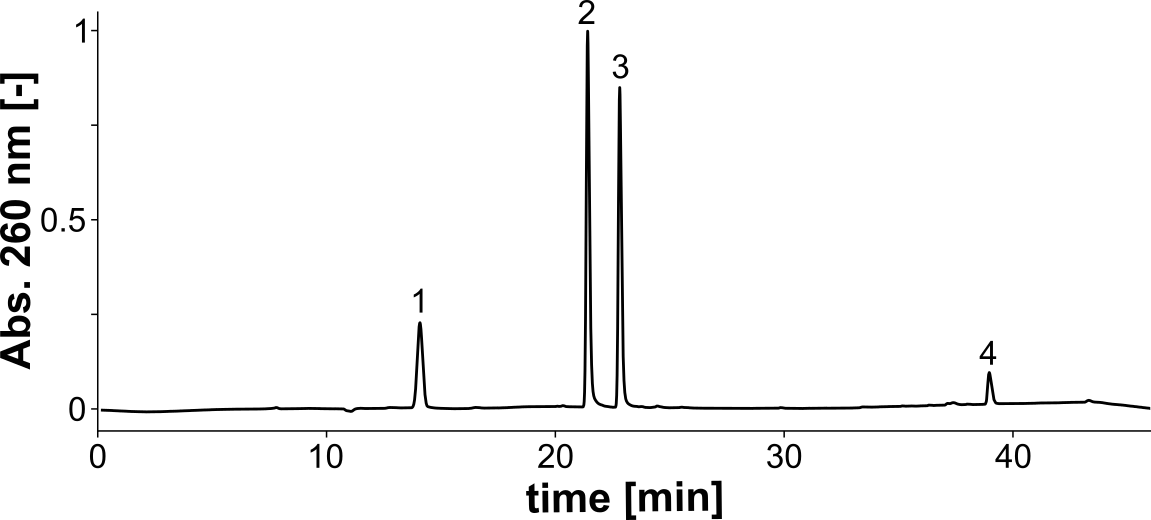


| Peak | Assignments | Molecule | Calc. Mass | Measured Mass |
| --- | --- | --- | --- | --- |
| 1 | dC | [M + H]^+^ | 228.22 | 228.22 |
| 2 | dA | [M + H]^+^ | 252.25 | 252.25 |
| 3 | dG | [M + H]^+^ | 268.25 | 268.25 |
| 4 | Ind-dU | [M + H]^+^ | 439.44 | 439.45 |

**Figure S21:** QC LC/MS CP5.47 Ind-dU


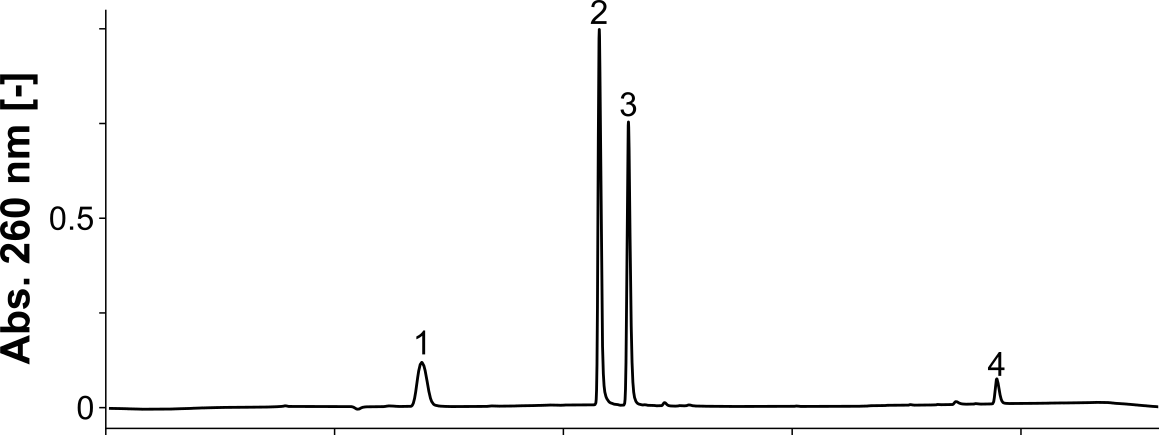


| Peak | Assignments | Molecule | Calc. Mass | Measured Mass |
| --- | --- | --- | --- | --- |
| 1 | dC | [M + H]^+^ | 228.22 | 228.20 |
| 2 | dA | [M + H]^+^ | 252.25 | 252.22 |
| 3 | dG | [M + H]^+^ | 268.25 | 268.21 |
| 4 | Ind-dU | [M + H]^+^ | 439.44 | 439.38 |

**Figure S22:** QC LC/MS CP5.47 scr Ind-dU


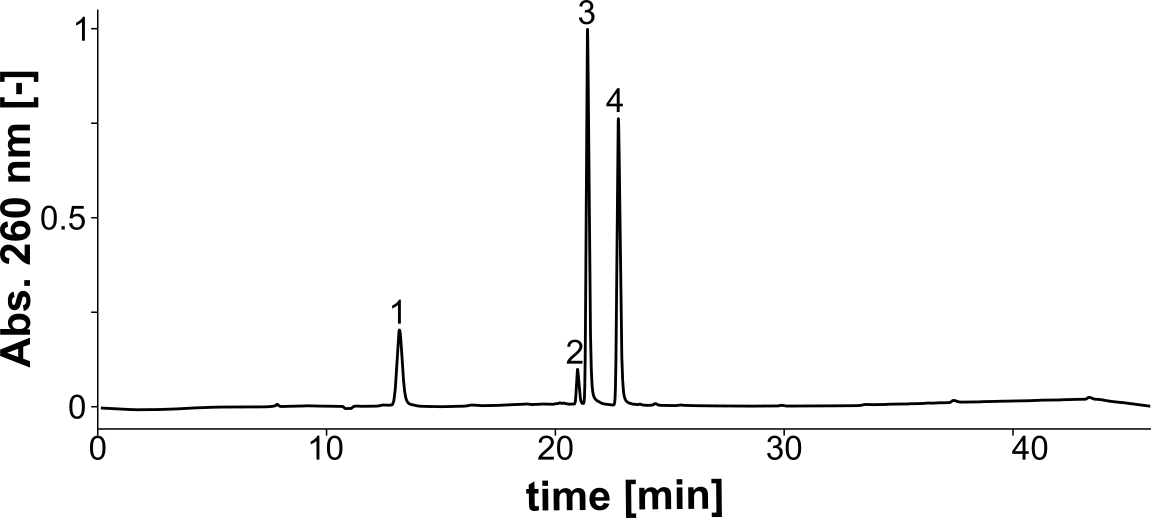


| Peak | Assignments | Molecule | Calc. Mass | Measured Mass |
| --- | --- | --- | --- | --- |
| 1 | dC | [M + H]^+^ | 228.22 | 228.22 |
| 2 | EA-dU | [M + H]^+^ | 339.33 | 339.32 |
| 3 | dA | [M + H]^+^ | 252.25 | 252.25 |
| 4 | dG | [M + H]^+^ | 268.25 | 268.25 |

**Figure S23:** QC LC/MS CP5.47 EA-dU


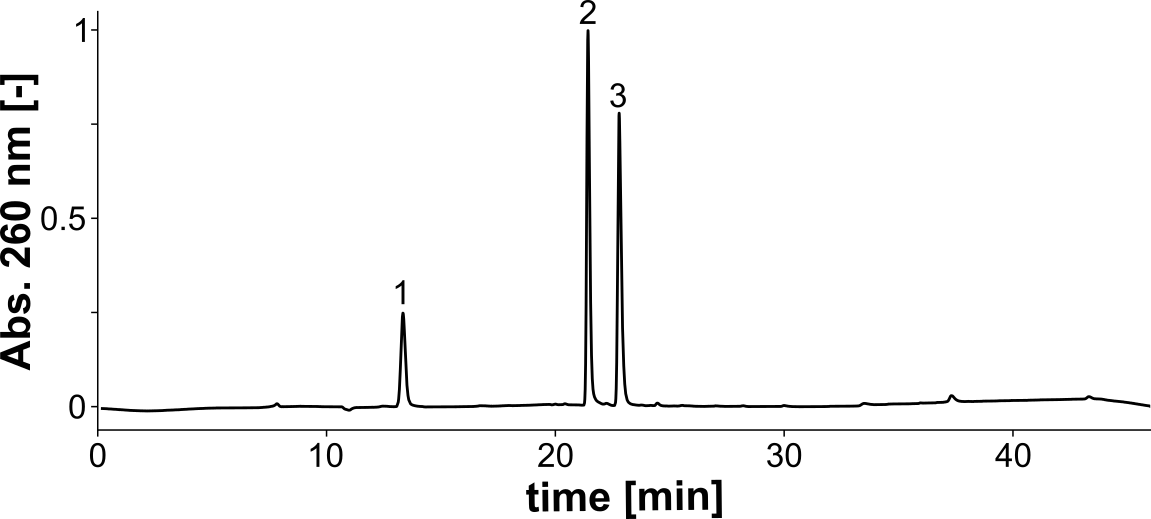


| Peak | Assignments | Molecule | Calc. Mass | Measured Mass |
| --- | --- | --- | --- | --- |
| 1 | dC | [M + H]^+^ | 228.22 | 228.22 |
| 2 | dA | [M + H]^+^ | 252.25 | 252.25 |
| 3 | dG  Imi-dU | [M + H]^+^  [M + H]^+^ | 268.25  390.37 | 268.25  390.37 |

**Figure S24:** QC LC/MS CP5.47 Imi-dU


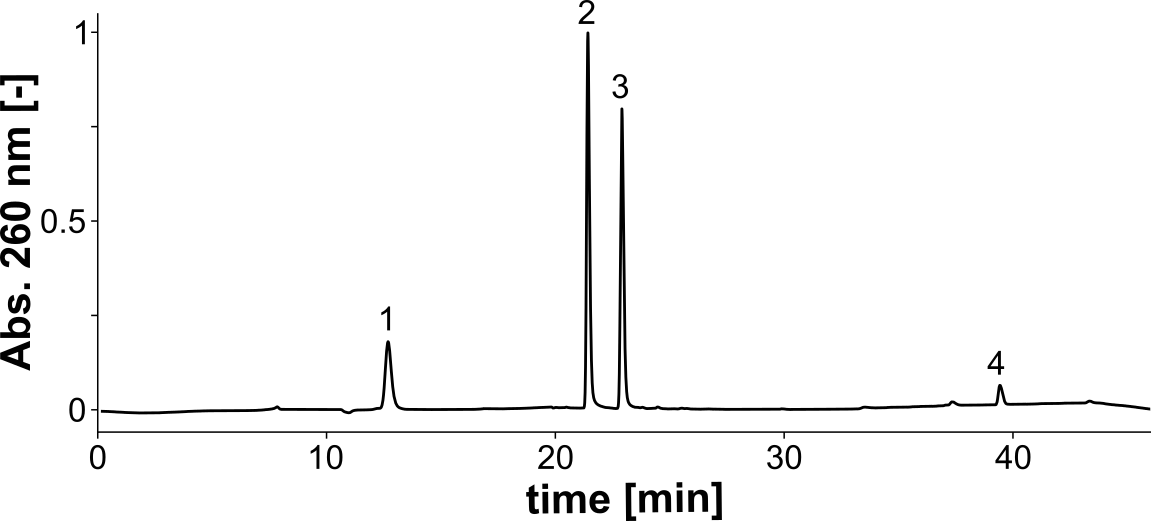


| Peak | Assignments | Molecule | Calc. Mass | Measured Mass |
| --- | --- | --- | --- | --- |
| 1 | dC | [M + H]^+^ | 228.22 | 228.22 |
| 2 | dA | [M + H]^+^ | 252.25 | 252.25 |
| 3 | dG | [M + H]^+^ | 268.25 | 268.25 |
| 4 | Tol-dU | [M + H]^+^ | 400.41 | 400.41 |

**Figure S25:** QC LC/MS CP5.47 Tol-dU


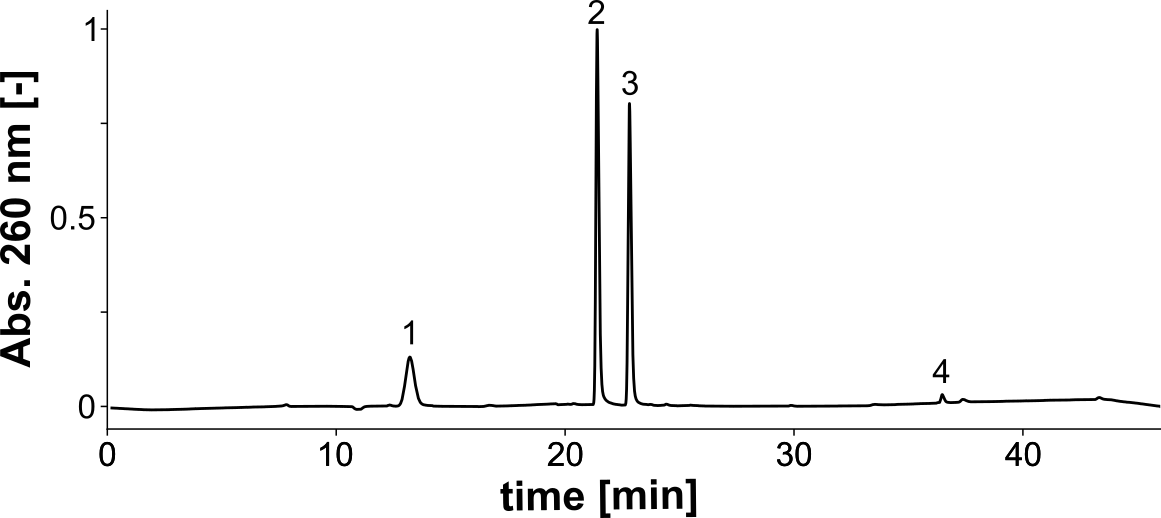


| Peak | Assignments | Molecule | Calc. Mass | Measured Mass |
| --- | --- | --- | --- | --- |
| 1 | dC | [M + H]^+^ | 228.22 | 228.22 |
| 2 | dA | [M + H]^+^ | 252.25 | 252.25 |
| 3 | dG | [M + H]^+^ | 268.25 | 268.25 |
| 4 | Phe-dU | [M + H]^+^ | 416.41 | 416.40 |

**Figure S26:** QC LC/MS CP5.47 Phe-dU


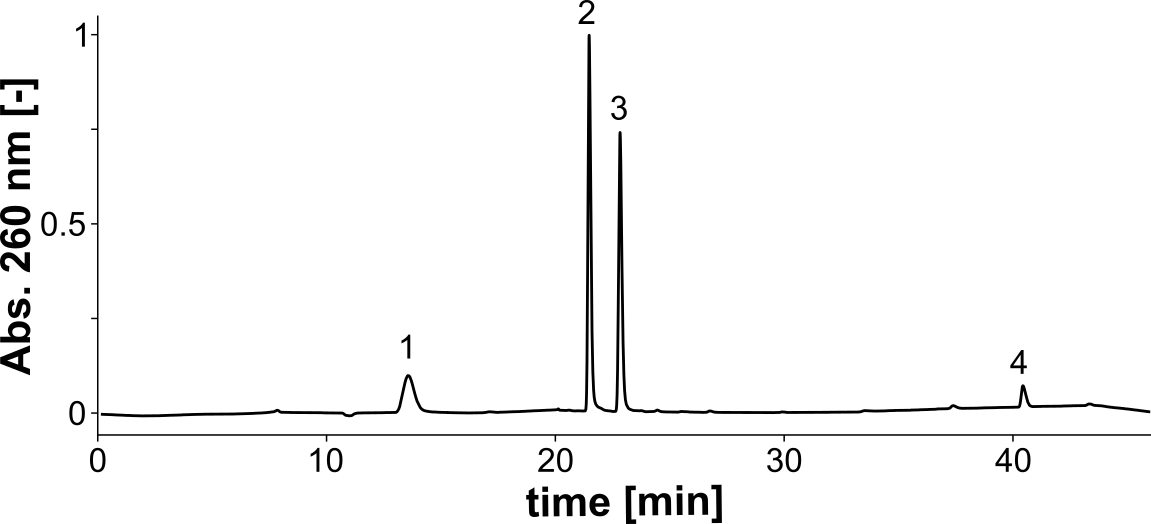


| Peak | Assignments | Molecule | Calc. Mass | Measured Mass |
| --- | --- | --- | --- | --- |
| 1 | dC | [M + H]^+^ | 228.22 | 228.22 |
| 2 | dA | [M + H]^+^ | 252.25 | 252.25 |
| 3 | dG | [M + H]^+^ | 268.25 | 268.25 |
| 4 | Nap-dU | [M + H]^+^ | 436.44 | 436.44 |

**Figure S27:** QC LC/MS CP5.47 Nap-dU

**
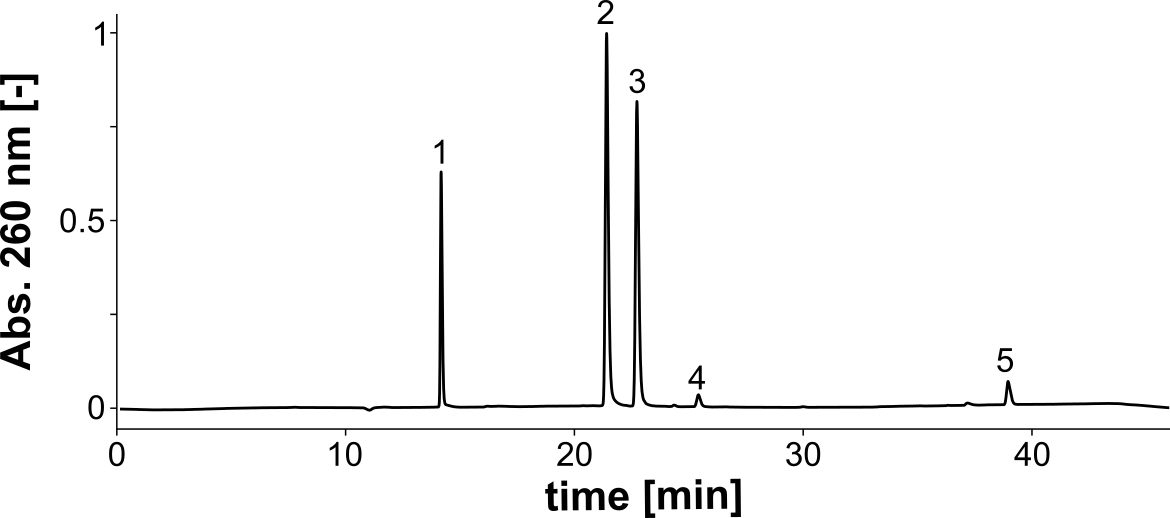
**

| Peak | Assignments | Molecule | Calc. Mass | Measured Mass |
| --- | --- | --- | --- | --- |
| 1 | dC | [M + H]^+^ | 228.22 | 228.22 |
| 2 | dA | [M + H]^+^ | 252.25 | 252.25 |
| 3 | dG | [M + H]^+^ | 268.25 | 268.25 |
| 4 | dT | [M + H]^+^ | 243.24 | 243.22 |
| 5 | Ind-dU | [M + H]^+^ | 439.44 | 439.45 |

**Figure S28:** QC LC/MS CP5.47 TXXX Ind-dU


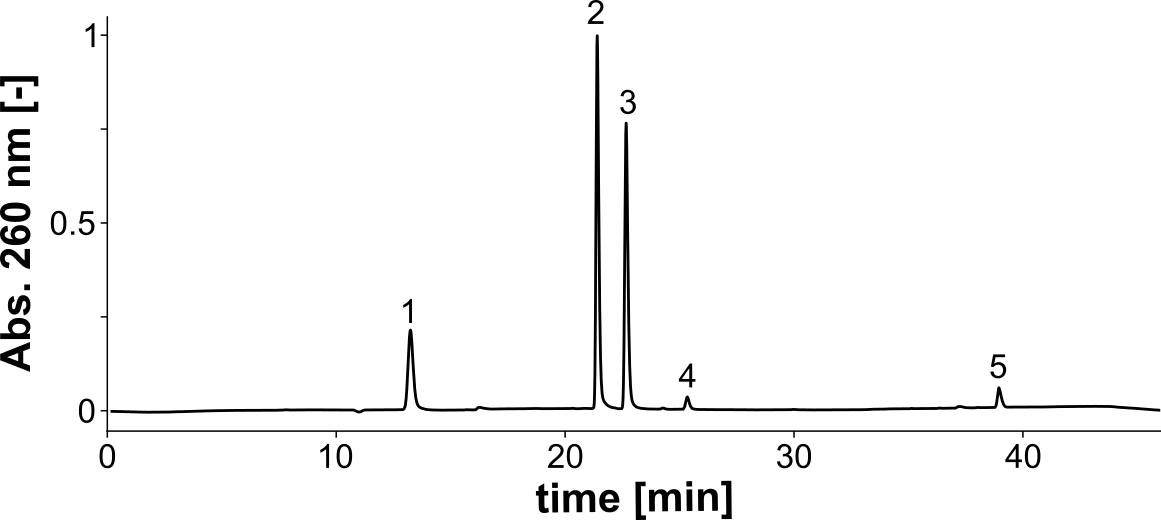


| Peak | Assignments | Molecule | Calc. Mass | Measured Mass |
| --- | --- | --- | --- | --- |
| 1 | dC | [M + H]^+^ | 228.22 | 228.22 |
| 2 | dA | [M + H]^+^ | 252.25 | 252.25 |
| 3 | dG | [M + H]^+^ | 268.25 | 268.25 |
| 4 | dT | [M + H]^+^ | 243.24 | 243.25 |
| 5 | Ind-dU | [M + H]^+^ | 439.44 | 439.45 |

**Figure S29:** QC LC/MS CP5.47 XTXX Ind-dU


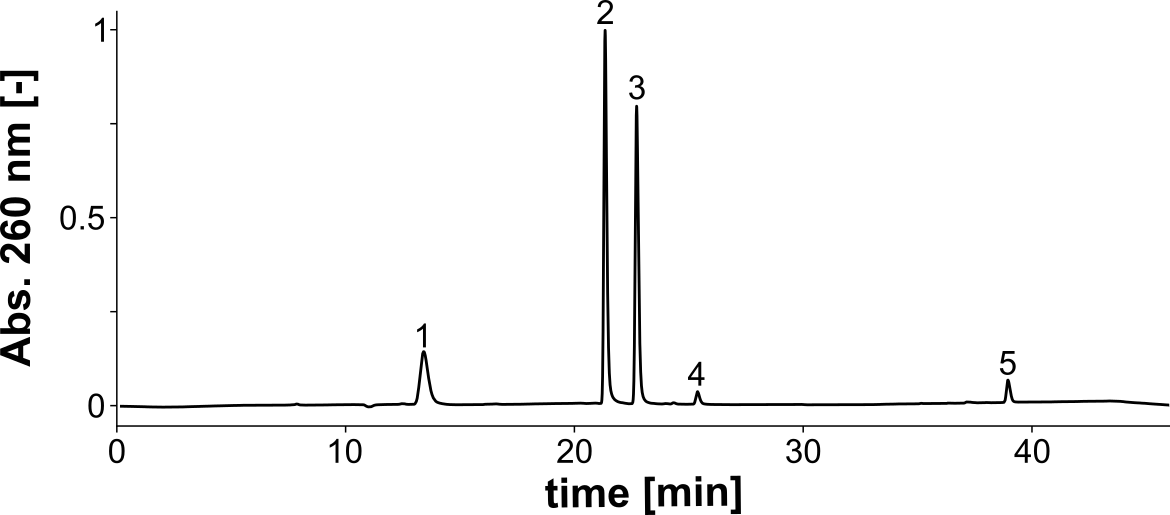


| Peak | Assignments | Molecule | Calc. Mass | Measured Mass |
| --- | --- | --- | --- | --- |
| 1 | dC | [M + H]^+^ | 228.22 | 228.23 |
| 2 | dA | [M + H]^+^ | 252.25 | 252.25 |
| 3 | dG | [M + H]^+^ | 268.25 | 268.25 |
| 4 | dT | [M + H]^+^ | 243.24 | 243.21 |
| 5 | Ind-dU | [M + H]^+^ | 439.44 | 439.44 |

**Figure S30:** QC LC/MS CP5.47 XXTX Ind-dU


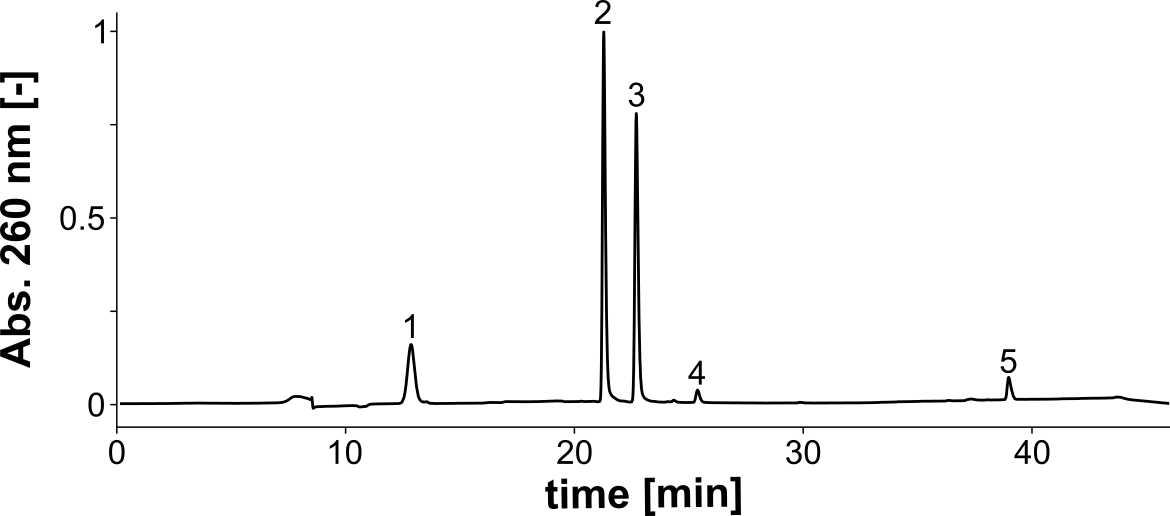


| Peak | Assignments | Molecule | Calc. Mass | Measured Mass |
| --- | --- | --- | --- | --- |
| 1 | dC | [M + H]^+^ | 228.22 | 228.22 |
| 2 | dA | [M + H]^+^ | 252.25 | 252.25 |
| 3 | dG | [M + H]^+^ | 268.25 | 268.24 |
| 4 | dT | [M + H]^+^ | 243.24 | 243.21 |
| 5 | Ind-dU | [M + H]^+^ | 439.44 | 439.44 |

**Figure S31:** QC LC/MS CP5.47 XXXT Ind-dU


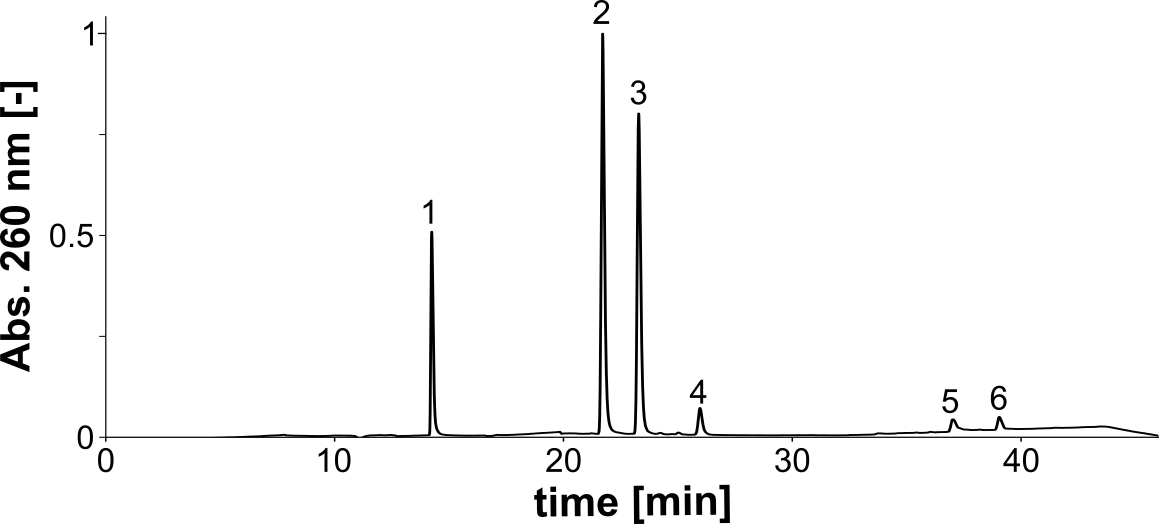


| Peak | Assignments | Molecule | Calc. Mass | Measured Mass |
| --- | --- | --- | --- | --- |
| 1 | dC | [M + H]^+^ | 228.22 | 228.22 |
| 2 | dA | [M + H]^+^ | 252.25 | 252.25 |
| 3 | dG | [M + H]^+^ | 268.25 | 268,24 |
| 4 | dT | [M + Na]^+^ | 265.22 | 265.22 |
| 5 | solvent | - | - | - |
| 6 | Ind-dU | [M + H]^+^ | 439.44 | 439.44 |

**Figure S32:** QC LC/MS CP5.47 XTXT Ind-dU


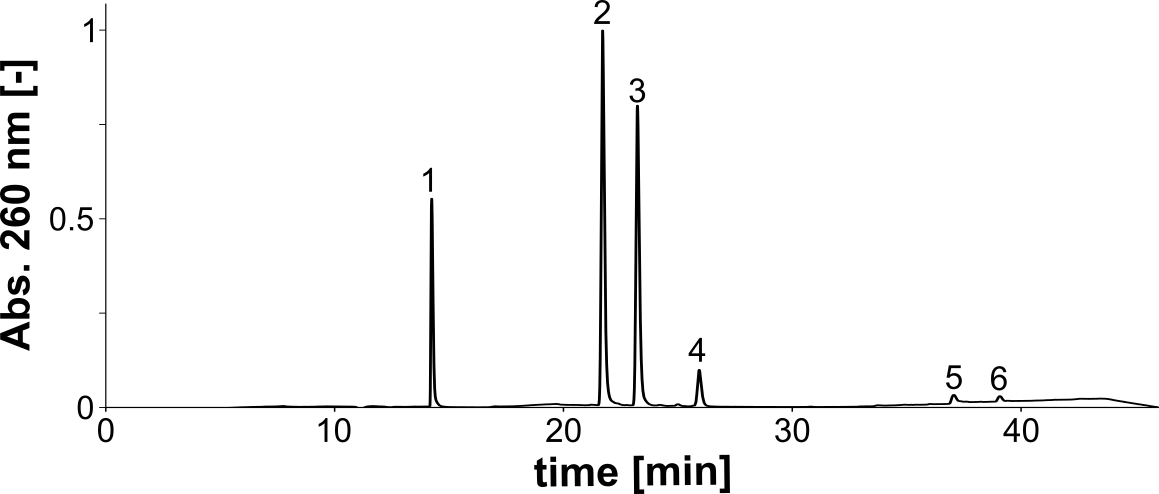


| Peak | Assignments | Molecule | Calc. Mass | Measured Mass |
| --- | --- | --- | --- | --- |
| 1 | dC | [M + H]^+^ | 228.22 | 228.22 |
| 2 | dA | [M + H]^+^ | 252.25 | 252.25 |
| 3 | dG | [M + H]^+^ | 268.25 | 268,24 |
| 4 | dT | [M + Na]^+^ | 265.22 | 265.24 |
| 5 | solvent | - | - | - |
| 6 | Ind-dU | [M + H]^+^ | 439.44 | 439.45 |

**Figure S33:** QC LC/MS CP5.47 TTXT Ind-dU


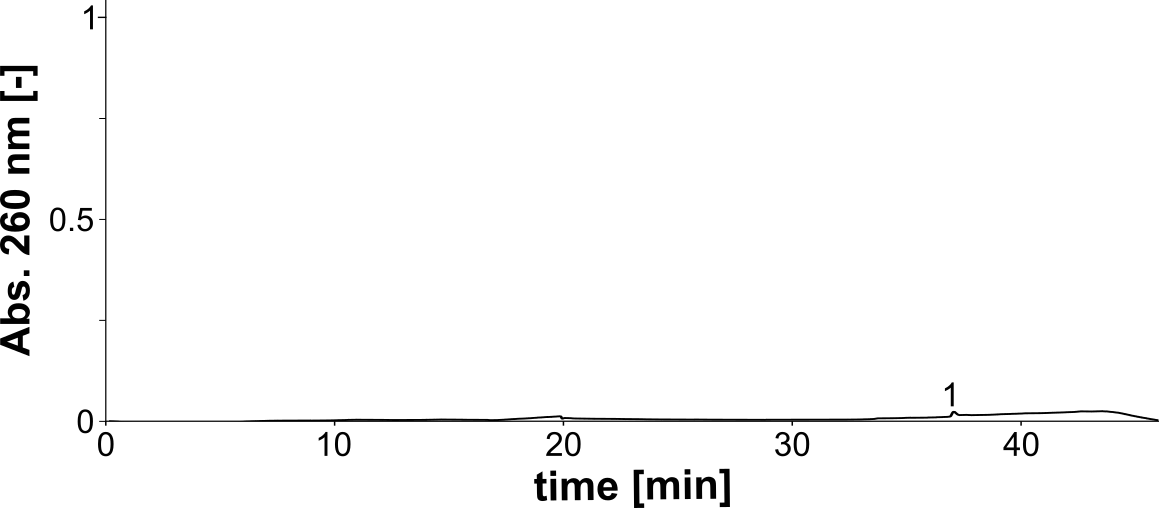


| Peak | Assignments | Molecule | Calc. Mass | Measured Mass |
| --- | --- | --- | --- | --- |
| 1 | solvent | - | - | - |

**Figure S34:** QC LC/MS solvent

**Table S2:** Kinetic parameters of the binding of CP-DS to CP-DS-Rv. The values are the mean and standard deviation of at least three independent experiments performed in duplicates.

| Modification | K_D_ [nM] | k_on_ [1/M*s] | k_off_ [1/s] |
| --- | --- | --- | --- |
| WT | 3.03 ± 0.35 | 7.86*10^4^ ± 0.4*10^4^ | 2.41*10^-4^ ± 0.35*10^-4^ |
| E-dU | 2.79 ± 1.00 | 7.78*10^4^ ± 1.55*10^4^ | 2.08*10^-4^ ± 0.52*10^-4^ |
| EA-dU | 7.25 ±1.74 | 8.54*10^4^ ± 0.99*10^4^ | 6.09*10^-4^ ± 1.04*10^-4^ |
| Imi-dU | 5.21 ± 1.03 | 1.27*10^5^ ± 0.4*10^4^ | 6.60*10^-4^ ± 1.35*10^-4^ |
| Tol-dU | 2.81 ± 1.11 | 1.39*10^5^ ± 5.5*10^4^ | 3.46*10^-4^ ± 0.8*10^-4^ |
| Phe-dU | 4.21 ± 1.16 | 9.97*10^4^ ± 0.36*10^4^ | 4.16*10^-4^ ± 0.99*10^-4^ |
| Ind-dU | 3.19 ± 1.21 | 1.40*10^5^ ± 2.9*10^4^ | 4.28*10^-4^ ± 1.31*10^-4^ |
| Nap-dU | 3.4 ± 0.59 | 1.53*10^5^ ± 0.5*10^4^ | 5.21*10^-4^ ± 0.91*10^-4^ |

**Table S3:** SELEX strategy

| SELEX Round | Target concentration [mM] | Target incubation time [min] | PCR cycles |
| --- | --- | --- | --- |
| 1 | 1 | 15 | 10 |
| 2 |  |  | 14 |
| 3 |  |  | 10 |
| 4 |  |  | 10 |
| 5 |  | 10 | 10 |
| 6 |  |  | 10 |
| 7 | 0.5 |  | 10 |
| 8 |  |  | 14 |
| 9 |  | 5 | 12 |
| 10 |  |  | 10 |


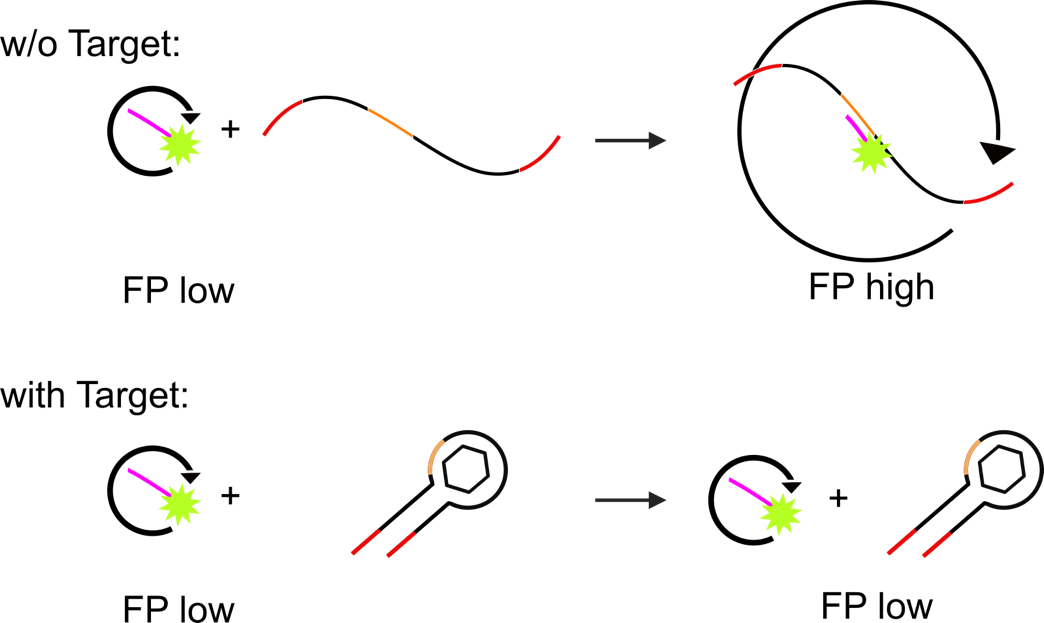


**Figure S35:** Schematic representation of a fluorescence polarization assay for binding assays.

**Table S4:** NGS Details

| SELEX Round | Total reads | Distinct sequences |
| --- | --- | --- |
| SL | 888386 | 813288 |
| 1 | 432363 | 397745 |
| 2 | 449869 | 407108 |
| 3 | 270671 | 247520 |
| 4 | 164753 | 149573 |
| 5 | 1334349 | 1196259 |
| 6 | 637615 | 583333 |
| 7 | 1084356 | 953762 |
| 8 | 1039814 | 839955 |
| 9 | 369654 | 296995 |
| 10 | 223859 | 153409 |

**
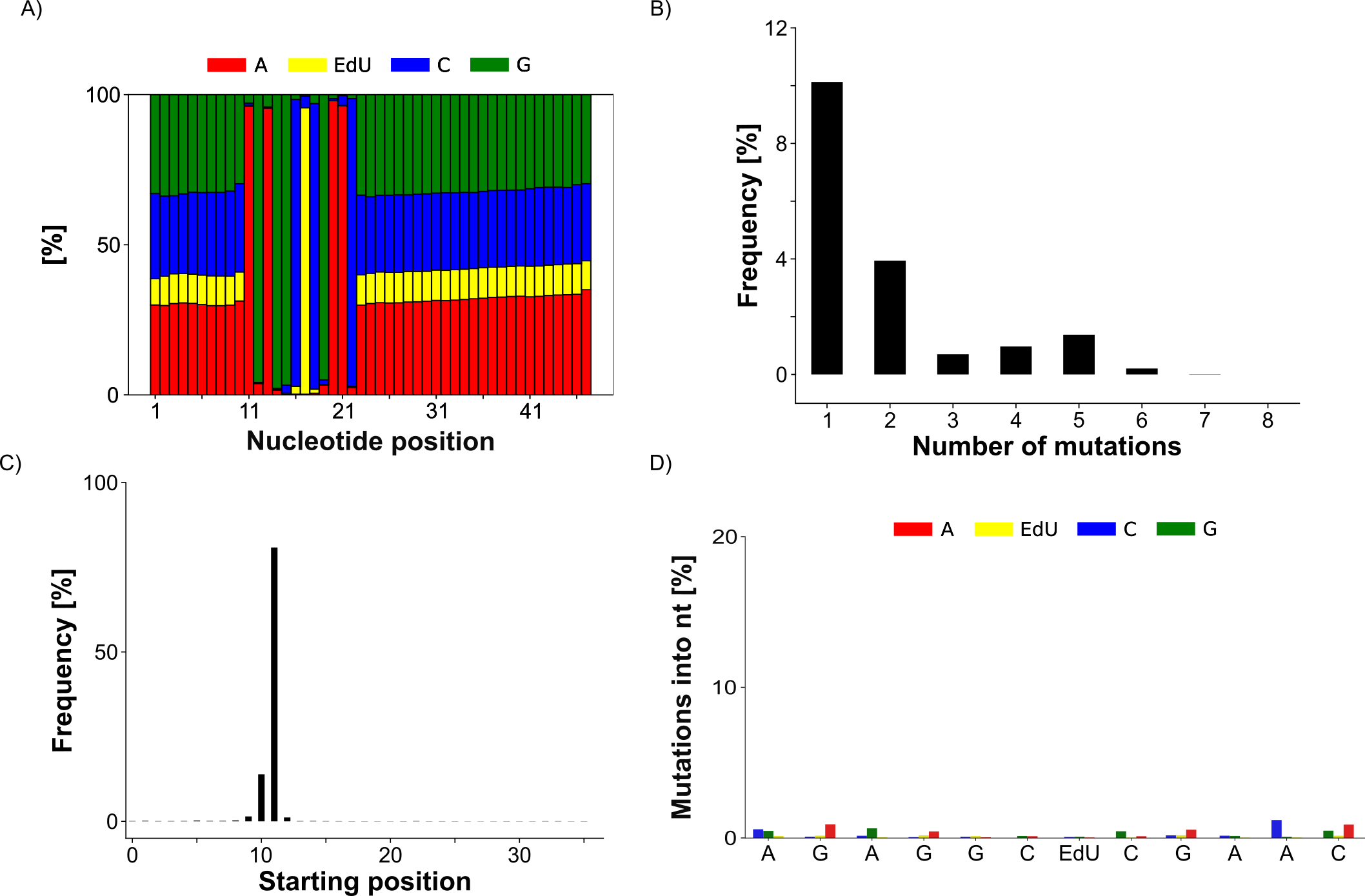
**

**Figure S36**: NGS analysis. A) Nucleotide distribution from the SL of CP-L. B) Quantification of the number of mutations in one docking sequence. C) Fraction of sequences with a starting position of the docking sequence at the indicated position. D) Mutation frequency with which mutations converted the original nucleotide from the docking sequence from the SL into the denoted nucleotide.

**Scheme S1:** Structures of kanamycin A, kanamycin B, tobramycin, gentamicin, D-(+)-glucose, paromomycin and ribostamycin. Kanamycin A differs from kanamycin B, tobramycin and gentamicin at the C2’’ position of one sugar moiety. This position is highlighted in red.


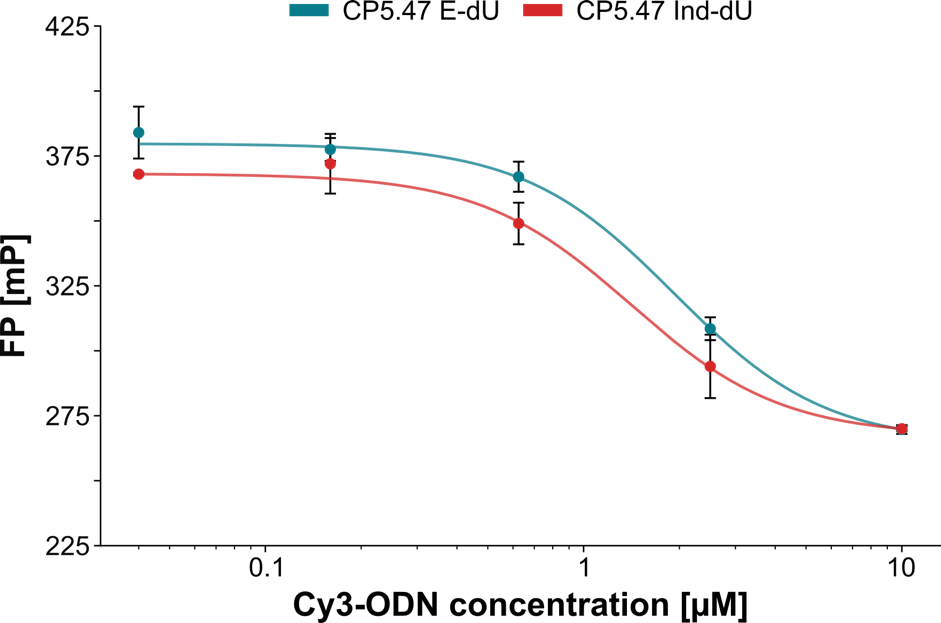


**Figure S37:** Fluorescence polarization (FP) assay to determine the K_D_ from the unmodified and indole-modified CP5.47 to the Cy3-ODN. The unmodified CP5.47 has a K_D_ of 1.9 ± 0.2 µM and the indole-modified CP5.47 has a K_D_ of 1.4 ± 0.4 µM. The K_D_ was determined by fitting a 4-parameter logistic function to the FP values at different Cy3-ODN concentrations.


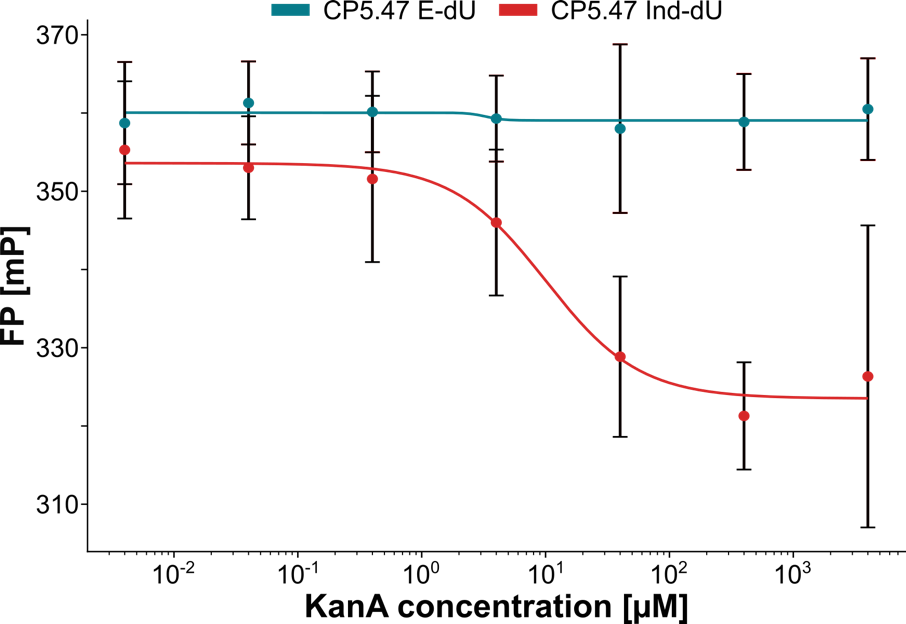


**Figure S38:** Fluorescence polarization (FP) assay to determine the K_D_ from the unmodified and indole-modified CP5.47 to kanamycin A. The K_D_ for the unmodified CP5.47 could not be determined, as no interaction between the sample and kanamycin A was detected. The KD of the indole-modified CP5.47 has a predicted K_D_ of 10 µM (R^2^ = 0.6; RSME = 10.3). The K_D_ was determined by fitting a 4-parameter logistic function to the FP values at different kanamycin A (KanA) concentrations (N = 3, at least in duplicates, Mean +- ci95%).

**Table S5:** Description of the ITC conditions

| Sequence | Cell conc. [µM] | K_D_ [µM] | ΔH [kcal/mol] | -TΔS [kcal/mol] | ΔG [kcal/mol] | N [sites] |
| --- | --- | --- | --- | --- | --- | --- |
| CP5.47 E-dU | 10 | n.a. | n.a. | n.a. | n.a. | n.a. |
| CP5.47 Ind-dU | 12 | 6 ± 3 | -20 ± 8 | 13 | -7 | 0.5 ± 0.1 |
| CP5.47 TTXT Ind-dU | 10 | n.a. | n.a. | n.a. | n.a. | n.a. |
| CP5.47 XTXT Ind-dU | 17 | 8 ± 4 | -19 ±4 | 12 | -7 | 1.0 ± 0.1 |

**Experimental section**

**Material**

The water and acetonitrile were purchased from VWR as MS-grade. The formic acid was purchased from Merck.

**Quality control of the click efficiency of the modified samples using LC-MS**

All modified sequences were tested for their click efficiency.

The sequences were enzymatically digested to single nucleosides using a nucleoside digestion mix from New England Biolabs, according to the instructions of the manufacturer. The nucleosides were analyzed on an Agilent 1100 HPLC system with an EC Nucleodur C18 gravity column (4.6 x 250 mm, 5 µm) from Macherey Nagel using a gradient of water (Solvent A) and acetonitrile (Solvent B), both supplemented with 0.1% formic acid, with a flow rate of 0.3 mL. The nucleosides were separated with the following linear gradient elution conditions (minutes/B%): 0/2, 3/2, 20/80, 34/30, 35/30, 35.01/98, 46/98. The chromatogram was recorded at 260 nm to identify the peaks. The sample containing the peaks was injected into a compact qToF from Bruker for compound identification. Data evaluation was performed using the Bruker Compass DataAnalysis Software (v 5.2). The absorption was normalized based on the highest peak intensity.
